# Modifying effect of hospital size on the impact of antimicrobial stewardship programs for methicillin-resistant *Staphylococcus aureus* bloodstream infections: a nationwide claims database analysis

**DOI:** 10.1186/s40780-026-00558-2

**Published:** 2026-02-26

**Authors:** Keisuke Sawada, Ryo Inose, Yuichi Muraki

**Affiliations:** 1 Department of Pharmacy, Federation of National Public Service Personnel Mutual Aid Associations Hirakata Kohsai Hospital, 1-2-1, Fujisaka Higashi-machi, Hirakata-shi, Osaka 573-0153 Japan; 2 Laboratory of Clinical Pharmacoepidemiology, Kyoto Pharmaceutical University, 5 Misasagi Nakauchi-cho, Yamashina-ku, Kyoto, 607-8414 Japan

**Keywords:** Antimicrobial stewardship, Methicillin-resistant *Staphylococcus aureus*, Bacteremia, Administrative claims, Treatment outcome, Costs and cost analysis, Effect modifier, Propensity score, Hospital bed capacity, Japan

## Abstract

**Background:**

The effectiveness of antimicrobial stewardship programs may vary by institutional context. We evaluated whether hospital size modifies the impact of nationally incentivized antimicrobial stewardship programs on clinical and economic outcomes in patients with methicillin-resistant *Staphylococcus aureus* bloodstream infections.

**Methods:**

This retrospective cohort study analyzed Japanese nationwide claims data from 2018 to 2022. We compared outcomes between adult inpatients with methicillin-resistant *Staphylococcus aureus* bloodstream infections in large (≥ 500 beds) versus non-large (< 500 beds) hospitals using 1:1 propensity score matching. The primary outcome was time to discharge. We used an interaction term to assess effect modification by hospital size on the association between antimicrobial stewardship programs and outcomes.

**Results:**

The matched cohort included 926 patients (463 pairs). At baseline, average daily total antimicrobial costs were significantly higher in large hospitals (rate ratio 1.32, 95% CI 1.16–1.50). Hospital size significantly modified the association between antimicrobial stewardship programs and time to discharge (P for interaction = 0.01). In non-large hospitals, antimicrobial stewardship programs were associated with significantly shorter time to discharge (hazard ratio 0.31, 95% CI 0.13–0.75). In large hospitals, antimicrobial stewardship programs were not associated with time to discharge but were associated with significantly reduced average daily antipseudomonal drug costs (rate ratio 0.46, 95% CI 0.30–0.71).

**Conclusions:**

Hospital size modified the association between antimicrobial stewardship program fee acquisition and outcomes among patients with methicillin-resistant *Staphylococcus aureus* bloodstream infections. In non-large hospitals, antimicrobial stewardship program fee acquisition was associated with shorter time to discharge, whereas in large hospitals, it was primarily associated with changes in prescribing patterns. These findings suggest that stewardship evaluation and policy should be tailored to institutional characteristics.

**Supplementary Information:**

The online version contains supplementary material available at 10.1186/s40780-026-00558-2.

## Background

Methicillin-resistant *Staphylococcus aureus* (MRSA) bloodstream infections (BSIs) represent a significant global health threat, with high mortality, prolonged hospitalization, and substantial healthcare costs [1–3]. Effective management of these infections requires timely and appropriate antimicrobial therapy combined with adequate source control [4–6]. However, treatment approaches and the resources available to implement them can vary considerably across different healthcare settings, potentially leading to significant disparities in patient outcomes and resource utilization [7]. 

In Japan, institutional characteristics, particularly hospital size, are key determinants of available resources [8]. Large hospitals (≥ 500 beds) typically function as advanced treatment centers with access to infectious disease specialists and comprehensive diagnostic capabilities. In contrast, non-large hospitals (< 500 beds) may operate with fewer specialized resources [9, 10]. Antimicrobial stewardship programs (ASPs) have been widely implemented to standardize care and promote the judicious use of antimicrobials across all settings. These ASPs are supported by a national financial incentive system introduced in 2018, with the incentive serving as a structural marker for facilities that have established a multidisciplinary ASP team and meet specific national criteria [8]. 

Previous studies have examined either the impact of hospital characteristics on infection outcomes or the general effectiveness of ASPs [11, 12]. However, whether these nationally incentivized ASPs demonstrate uniform effectiveness across institutions of varying size and resource availability remains unknown. Understanding the interaction between hospital size and ASP implementation is crucial for optimizing MRSA BSI management, tailoring stewardship strategies to specific institutional contexts, and ensuring equitable care delivery on a national scale.

Therefore, this study investigated whether hospital size modifies the impact of ASPs on clinical and economic outcomes in patients with MRSA BSI. We compared clinical outcomes and antimicrobial use between patients with MRSA BSIs in large versus non-large hospitals using a nationwide Japanese claims database.

## Methods

### Ethics

This study utilized a pre-existing, fully anonymized dataset classified as “already created anonymized information” under Japanese ethical guidelines, which exempted it from formal ethical review requirements. We obtained official permission from Japan Medical Data Center (JMDC) Inc. to access and analyze the database. The Kyoto Pharmaceutical University Ethics Committee confirmed that formal ethical review was not required (exemption number: NR-00006). The requirement for individual informed consent was waived because the database consists of fully anonymized data.

The study was conducted in compliance with the principles of the Declaration of Helsinki. This study was performed in accordance with the Strengthening the Reporting of Observational Studies in Epidemiology (STROBE), RECORD (REporting of studies Conducted using Observational Routinely-collected health Data), and RECORD for Pharmacoepidemiologic Research (RECORD-PE) guidelines [13–15]. 

### Study design and data source

This retrospective cohort study employed a comparative effectiveness research design using propensity score matching to control for confounding. We analyzed data from the JMDC institutional database. The JMDC database comprises data collected directly from a nationwide network of contracted hospitals in Japan [16, 17]. It contains comprehensive inpatient data, including administrative claims information and Diagnosis Procedure Combination (DPC) survey data. The DPC data include detailed clinical information from discharge summaries and electronic claims files detailing procedures, medications, and devices [18, 19]. A key feature of this hospital-based database is its high proportion of elderly patients (approximately 42% aged ≥ 65 years) [16], making it highly suitable for studying conditions such as MRSA BSI. Furthermore, the database includes both DPC-participating and non-DPC hospitals, and its distribution by hospital bed scale closely mirrors the national average, ensuring high generalizability to real-world clinical practice in Japan [17]. 

### Study setting, period, and population

The study period spanned April 2018 to March 2022. This period was chosen to align with the implementation of Japan’s national Antimicrobial Stewardship Fee introduced in April 2018, enabling examination of this policy’s impact. We identified patients hospitalized with MRSA BSI using an algorithm that combined a primary or principal diagnosis of MRSA sepsis (Standard Disease Code 8830124) with pharmacy claims for anti-MRSA therapy. This algorithm has demonstrated high positive predictive value for identifying true MRSA BSI in administrative data [20, 21]. To ensure a comparable baseline of infection control infrastructure across the study cohort, we included patients only if they were treated at facilities claiming Infection Prevention Fee 1. This reimbursement fee, which serves as a structural marker for hospitals with an established multidisciplinary infection control team, is a prerequisite for claiming the Antimicrobial Stewardship Fee. We excluded patients if they (1) had missing claims data; (2) received no anti-MRSA drugs; (3) were transferred from another hospital; (4) were transferred to another hospital; or (5) had missing components of the SOFA score [22]. Regarding missing claims data, the JMDC database aggregates institutional claims data on a monthly basis. Therefore, patients whose hospitalization spanned months during which the facility did not submit complete data had incomplete hospitalization records.

### Variables

We defined and extracted all variables from the JMDC claims data (Supplementary Tables 1–3).

#### Primary exposure

The primary exposure was hospital size, categorized as “large hospitals” (≥ 500 beds) and “non-large hospitals” (< 500 beds). This classification aligns with the Japanese healthcare system, where hospitals with ≥ 500 beds typically function as legally designated “Special Function Hospitals” or “Regional Medical Care Support Hospitals” with comprehensive medical capabilities [23]. 

#### Effect modifier

The effect modifier was acquisition of the Antimicrobial Stewardship Fee (procedure code: 190206870) during hospitalization. This fee indicates the presence of an active, multidisciplinary ASP that meets specific national criteria for stewardship activities.

#### Outcome measure

The primary outcome was time from MRSA BSI onset to discharge. MRSA BSI onset (day 0) was operationally defined as the date of the initial SOFA score measurement. The SOFA score is systematically recorded on the day of a sepsis diagnosis in Japan’s DPC system [24, 25]. The secondary outcomes were all-cause mortality within 30 days of MRSA BSI onset and unplanned readmission within 30 days of discharge. Unplanned readmissions were identified based on the readmission classification in the Form 1 file of the database, by excluding admissions explicitly coded as “planned.” Antimicrobial treatment duration and average daily antimicrobial costs were also assessed.

For antimicrobial treatment duration, the total number of days a patient received at least one antimicrobial agent was calculated. The concurrent use of multiple agents on a single day was counted as one day, and for oral medications, the duration was based on the number of days supplied from the prescription date. Total antimicrobial costs were calculated based on the official drug price at the time of administration. The average daily cost was then derived by dividing the total cost by the number of days in the respective analysis period. These outcomes of treatment duration and cost were analyzed for two distinct periods: (1) from MRSA BSI onset to discharge (primary analysis) and (2) over the entire hospitalization period (sensitivity analysis). For both periods, the outcomes were further categorized by major drug classes (total antimicrobials, anti-MRSA agents, antipseudomonal agents, other antimicrobials, antifungals, and antivirals) and by route of administration (injectable and oral).

#### Covariates

We selected covariates for propensity score matching based on previous literature and clinical guidelines [26, 27]. These included patient demographics (sex, age, care facility residence, hospital admission within 90 days); clinical characteristics and interventions (infective endocarditis, surgery, central venous catheter placement, dialysis, SOFA score); and comorbidities, including myocardial infarction, congestive heart failure, peripheral vascular disease, cerebrovascular disease, dementia, chronic pulmonary disease, rheumatic disease, peptic ulcer disease, mild liver disease, diabetes without and with complications, hemiplegia or paraplegia, renal disease, any malignancy, moderate/severe liver disease, metastatic solid tumor, and acquired immunodeficiency syndrome/human immunodeficiency virus infection. All comorbidities were identified using the Quan modification of the Charlson Comorbidity Index (CCI) [28]. 

### Statistical analysis

Before conducting primary analysis, we verified the proportional hazards assumption for Cox models, including the fully specified models with interaction terms, using visual inspection of log-log survival plots and Schoenfeld residuals [29]. All statistical analysis was conducted using the survival and glmmTMB packages in R version 4.1.0 (R Foundation for Statistical Computing, Vienna, Austria). Statistical significance was defined as a two-sided p value of < 0.05.

#### Propensity score matching

To balance baseline characteristics, we performed 1:1 nearest-neighbor propensity score matching without replacement using a caliper width of 0.2 standard deviations of the logit of the propensity score. The propensity score, representing the probability of being treated in a large hospital, was estimated using a multivariable logistic regression model that included all predefined covariates. Matching quality was assessed using standardized mean differences (SMDs), with an SMD < 0.1 considered to indicate adequate balance.

#### Main analysis

After matching, we compared outcomes between the two hospital size groups. For the primary outcome measure of time to discharge, a stratified Cox proportional hazards regression model was used. For binary outcomes (30-day mortality and 30-day readmission), conditional logistic regression was used. For antimicrobial treatment duration (count data), we used negative binomial generalized linear mixed models (GLMMs) with a log link. For antimicrobial costs, which were characterized by zero-inflated and right-skewed distributions, the average daily cost was analyzed using Tweedie GLMMs with a log link [30]. All mixed models included a random intercept for each matched pair to account for the paired-data structure.

#### Effect modification analysis

To evaluate whether hospital size modified the effect of ASP fee acquisition, we introduced a multiplicative interaction term (hospital size × ASP fee acquisition) into the respective statistical models for each outcome [31]. Stratum-specific effects of ASPs within large and non-large hospitals were then estimated from these interaction models.

## Results

### Patient characteristics and cohort comparability

**Fig. 1:**
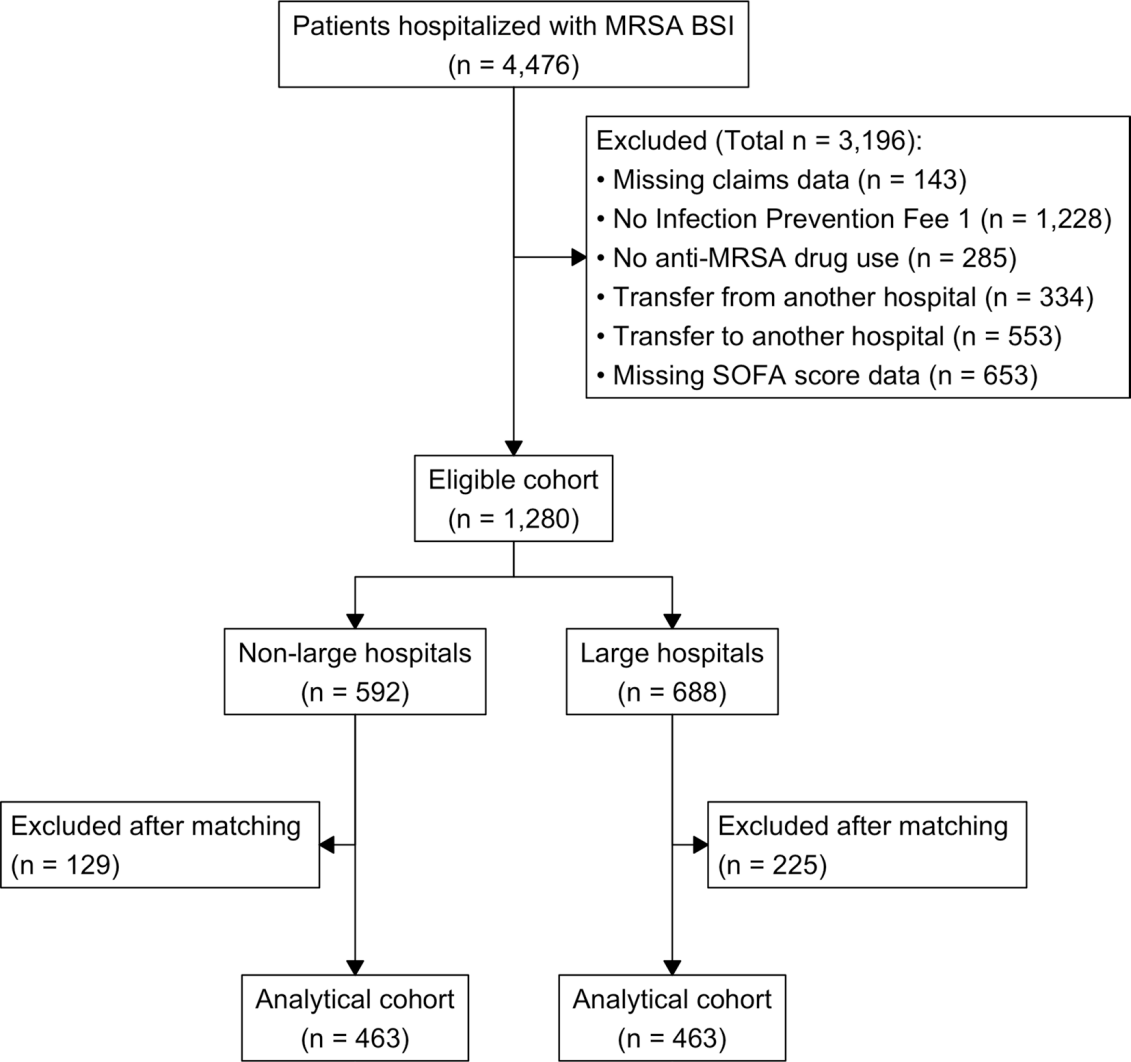
Flow diagram of patient selection. Abbreviations: MRSA BSI, methicillin-resistant *Staphylococcus aureus* bloodstream infection; SOFA, Sequential Organ Failure Assessment

Among 4,476 patients initially identified, the final eligible cohort included 1,280 patients: 592 (46.3%) treated in non-large hospitals and 688 (53.7%) treated in large hospitals. Propensity score matching yielded 463 matched pairs (926 patients total) for the final analysis. Figure 1 shows the patient selection flowchart. Table 1 presents baseline patient characteristics before and after propensity score matching. The groups showed significant imbalances before matching. For instance, the large hospital group had higher prevalence of malignancy, while the non-large hospital group was older and had a higher proportion of care facility residents. All measured covariates were well balanced after propensity score matching, with all SMDs below the prespecified threshold of 0.1.

**Table 1:** Baseline patient characteristics stratified by hospital size before and after propensity score matching

Variable	Before matching			After matching		
	Non-large hospitals (*n* = 592)	Large hospitals (*n* = 688)	SMD	Non-large hospitals (*n* = 463)	Large hospitals (*n* = 463)	SMD
Demographics						
Male sex	384 (64.9%)	426 (61.9%)	0.06	290 (62.6%)	288 (62.2%)	0.01
Age, years	78 [69–86]	74 [64–82]	0.31	78 [67–85]	76 [69–84]	0.01
Care facility resident	87 (14.7%)	52 (7.6%)	0.23	56 (12.1%)	49 (10.6%)	0.05
Hospital admission within 90 days	150 (25.3%)	192 (27.9%)	0.06	110 (23.8%)	130 (28.1%)	0.09
Procedures and interventions						
Infective endocarditis	15 (2.5%)	21 (3.1%)	0.03	12 (2.6%)	15 (3.2%)	0.04
Surgery	87 (14.7%)	66 (9.6%)	0.16	64 (13.8%)	61 (13.2%)	0.02
Central venous catheter placement	256 (43.2%)	338 (49.1%)	0.12	206 (44.5%)	198 (42.8%)	0.04
Dialysis	103 (17.4%)	87 (12.7%)	0.13	70 (15.1%)	74 (16.0%)	0.02
Comorbidities and severity						
Charlson Comorbidity Index	2 [1–4]	2 [1–4]	0.04	2 [1–3]	2 [1–4]	0.05
SOFA score	4 [2–7]	4 [2–7]	0.08	4 [2–7]	4 [2–7]	0.04
Comorbidities by CCI components						
Myocardial infarction	19 (3.2%)	24 (3.5%)	0.02	17 (3.7%)	17 (3.7%)	0.00
Congestive heart failure	146 (24.7%)	135 (19.6%)	0.12	109 (23.5%)	112 (24.2%)	0.02
Peripheral vascular disease	37 (6.2%)	31 (4.5%)	0.08	24 (5.2%)	30 (6.5%)	0.06
Cerebrovascular disease	101 (17.1%)	93 (13.5%)	0.10	77 (16.6%)	73 (15.8%)	0.02
Dementia	85 (14.4%)	50 (7.3%)	0.23	52 (11.2%)	48 (10.4%)	0.03
Chronic pulmonary disease	25 (4.2%)	37 (5.4%)	0.05	18 (3.9%)	22 (4.8%)	0.04
Rheumatic disease	21 (3.5%)	28 (4.1%)	0.03	18 (3.9%)	18 (3.9%)	0.00
Peptic ulcer disease	44 (7.4%)	51 (7.4%)	0.00	30 (6.5%)	36 (7.8%)	0.05
Mild liver disease	28 (4.7%)	44 (6.4%)	0.07	24 (5.2%)	21 (4.5%)	0.03
Diabetes without complications	110 (18.6%)	120 (17.4%)	0.03	84 (18.1%)	85 (18.4%)	0.01
Diabetes with complications	53 (9.0%)	45 (6.5%)	0.09	38 (8.2%)	36 (7.8%)	0.02
Hemiplegia or paraplegia	5 (0.8%)	5 (0.7%)	0.01	4 (0.9%)	5 (1.1%)	0.02
Renal disease	138 (23.3%)	107 (15.6%)	0.20	94 (20.3%)	95 (20.5%)	0.01
Any malignancy	151 (25.5%)	330 (48.0%)	0.48	142 (30.7%)	146 (31.5%)	0.02
Moderate/severe liver disease	7 (1.2%)	15 (2.2%)	0.08	6 (1.3%)	5 (1.1%)	0.02
Metastatic solid tumor	41 (6.9%)	40 (5.8%)	0.05	27 (5.8%)	34 (7.3%)	0.06
AIDS/HIV	0 (0.0%)	3 (0.4%)	0.09	0 (0.0%)	0 (0.0%)	NA

Data are presented as n (%) for categorical variables and as the median [interquartile range] for continuous variables

The SMD is used to assess the balance of covariates after matching, with a value < 0.1 indicating a negligible difference between groups

Abbreviations: AIDS/HIV, acquired immunodeficiency syndrome/human immunodeficiency virus; CCI, Charlson Comorbidity Index; SMD, standardized mean difference; SOFA, Sequential Organ Failure Assessment

### Baseline comparisons of clinical outcomes, antimicrobial use, and antimicrobial cost

For the primary clinical outcome measure, although the median time from MRSA BSI onset to discharge was longer in non-large hospitals than in large hospitals (34 days vs. 29 days), this difference did not reach statistical significance (hazard ratio 0.83, 95% CI 0.65–1.07, *P* = 0.15). Similarly, no significant differences were observed for the secondary outcome measures of 30-day mortality (odds ratio 0.83, 95% CI 0.61–1.12, *P* = 0.22) and 30-day readmission (OR 1.02, 95% CI 0.67–1.58, *P* = 0.91) (Table 2).

Meanwhile, significant differences emerged in the duration of antimicrobial therapy and associated costs (Table 3). The total duration of antimicrobial therapy from onset did not differ significantly between non-large and large hospitals (median 18 days vs. 19 days, rate ratio 1.04, 95% CI 0.94–1.15). Similarly, no significant differences were observed for the duration of anti-MRSA drug therapy, total injectable agents, or total oral agents. However, the average daily total antimicrobial cost from MRSA BSI onset to discharge was 32% higher in large hospitals than in non-large hospitals (RR 1.32, 95% CI 1.16–1.50, *P* < 0.01). This cost difference was primarily driven by significantly higher costs for several specific drug classes, including antipseudomonal drugs (RR 1.18, 95% CI 1.00–1.39), other antimicrobials (RR 1.31, 95% CI 1.05–1.65), and antifungal drugs (RR 2.43, 95% CI 1.51–3.89) in large hospitals (Table 3). A further breakdown by route of administration revealed that the higher costs for antipseudomonal and other antimicrobial drugs were attributable to injectable formulations only. For antifungal drugs, the costs were significantly higher for both injectable and oral formulations (Supplementary Table 4).

**Table 2:** Comparison of clinical outcomes between patients in non-large and in large hospitals in the propensity score-matched cohort

Outcome	Non-large hospitals (*n* = 463)	Large hospitals (*n* = 463)	Effect measure	*P*-Value
Days from onset to discharge	34 [22–55]	29 [18–48]	HR, 0.83 [95% CI, 0.65–1.07]	0.15
30-Day mortality	122 (26.3%)	106 (22.9%)	OR, 0.83 [95% CI, 0.61–1.12]	0.22
30-Day readmission rate	43 (9.3%)	44 (9.5%)	OR, 1.02 [95% CI, 0.67–1.58]	0.91

Data are presented as n (%) or the median [interquartile range]

HRs are estimated using a stratified Cox proportional hazards regression model, conditioning on matched pairs. An HR < 1 indicates shorter time to discharge (higher rate of discharge) for patients in large hospitals. ORs for 30-day mortality and 30-day readmission are estimated using conditional logistic regression, conditioning on matched pairs

**Table 3:** Comparison of antimicrobial therapy duration and average daily costs from onset to discharge between patients in non-large and in large hospitals in the propensity score-matched cohort

Outcome	Non-large hospitals (*n* = 463)	Large hospitals (*n* = 463)	Effect measure, RR [95% CI]	*P*-Value
Duration of antimicrobial therapy (days)				
Total antimicrobial treatment	18 [10–32]	19 [11–33]	1.04 [0.94–1.15]	0.49
Anti-MRSA drug treatment	9 [4–16]	9 [5–16]	0.96 [0.86–1.07]	0.47
Injectable antimicrobial treatment	15 [9–26]	17 [10–28]	1.05 [0.95–1.16]	0.30
Oral antimicrobial treatment	0 [0–7]	0 [0–9]	0.98 [0.71–1.35]	0.88
Average daily antimicrobial costs (JPY/day)				
Total antimicrobial costs	1,787 [897–3,907]	2,133 [1,048–4,859]	1.32 [1.16–1.50]	< 0.01
Anti-MRSA drug costs	649 [343–1,697]	843 [345–1,870]	1.12 [0.96–1.29]	0.14
Antipseudomonal drug costs	358 [0–1,061]	359 [0–1,200]	1.18 [1.00–1.39]	0.047
Other antimicrobial costs	13 [0–159]	30 [0–259]	1.31 [1.05–1.65]	0.02
Antifungal drug costs	0 [0–0]	0 [0–0]	2.43 [1.51–3.89]	< 0.01
Antiviral drug costs	0 [0–0]	0 [0–0]	1.62 [0.62–4.22]	0.32

Data are presented as the median [interquartile range]

RRs for antimicrobial duration are estimated using negative binomial generalized linear mixed models. RRs for average daily costs are estimated using Tweedie generalized linear mixed models to account for zero-inflated and right-skewed cost distributions. A statistically significant p-value for cost categories with a median of 0 (e.g. antifungal drugs) indicates a significant difference in the frequency or magnitude of non-zero costs between the groups

Abbreviations: JPY, Japanese Yen; RR, rate ratio

### Effect modification of ASPs by hospital size

**Fig. 2:**
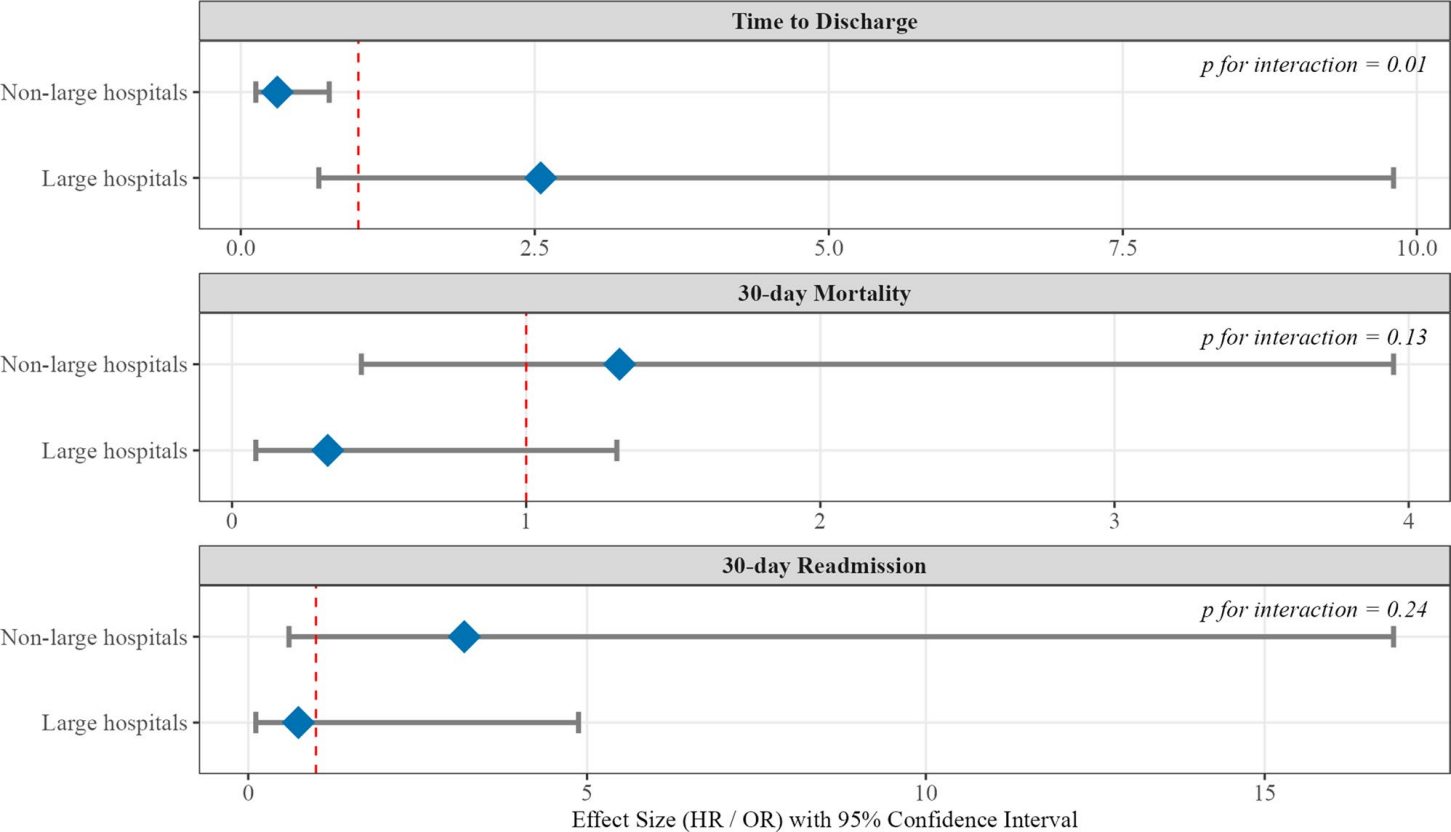
Effect modification by hospital size on the association between antimicrobial stewardship program (ASP) fee acquisition and clinical outcomes. Forest plot showing the association of ASP fee acquisition with clinical outcomes stratified by hospital size in the propensity score-matched cohort (*n* = 926). The reference group is patients in hospitals without ASP fee acquisition. For time to discharge, hazard ratios (HRs) are presented. An HR < 1 indicates shorter time to discharge associated with ASP acquisition. For mortality and readmission, odds ratios (ORs) are presented. Abbreviations: ASP, antimicrobial stewardship program

**Fig. 3:**
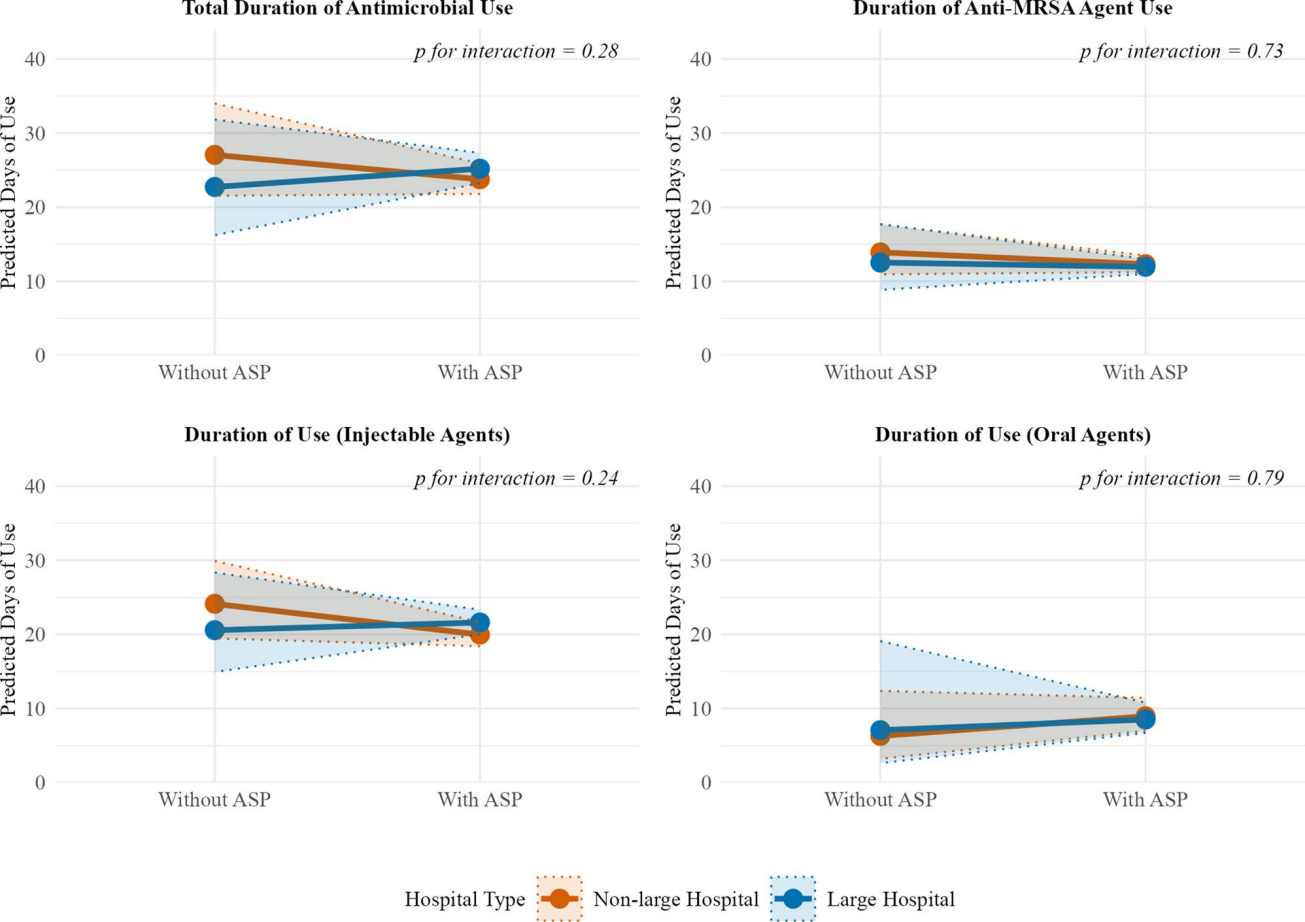
Effect modification by hospital size on the association between antimicrobial stewardship program (ASP) fee acquisition and antimicrobial therapy duration. Predicted durations of antimicrobial therapy in the propensity score-matched cohort (*n* = 926) from negative binomial generalized linear mixed models illustrating the interaction between hospital size and ASP fee acquisition. Abbreviations: ASP, antimicrobial stewardship program

Our analysis revealed that the impact of ASPs differed across outcomes. Hospital size significantly modified the association between ASP fee acquisition and time to discharge (P for interaction = 0.01; Fig. 2). In non-large hospitals, ASP fee acquisition was associated with significantly shorter time to discharge (HR 0.31, 95% CI 0.13–0.75), whereas in large hospitals, it was associated with a non-significant trend toward longer time to discharge (HR 2.56, 95% CI 0.66–10.0). In contrast, hospital size did not significantly modify the association between ASP fee acquisition and 30-day mortality (P for interaction = 0.13) or 30-day readmission (P for interaction = 0.24). Similarly, for antimicrobial therapy duration, hospital size did not significantly modify the association between ASP fee acquisition and duration for total antimicrobials, anti-MRSA drugs, injectable agents, or oral agents (Fig. 3). Furthermore, although not statistically significant, there was a trend toward longer oral antimicrobial therapy duration in hospitals with an ASP compared with those without an ASP in both non-large and large hospital strata (P for interaction = 0.79).

**Fig. 4:**
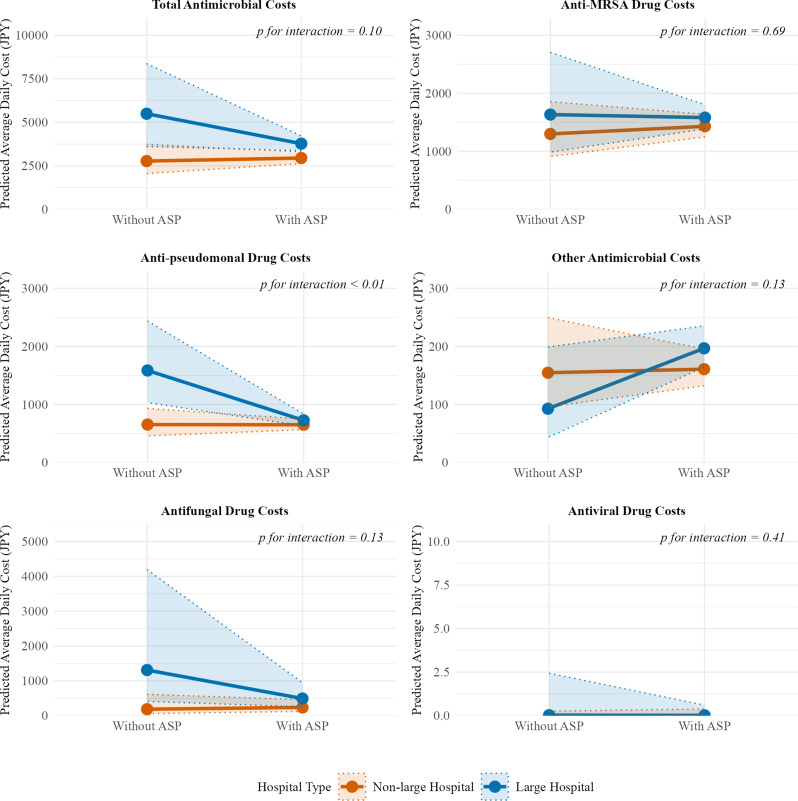
Effect modification by hospital size on the association between antimicrobial stewardship program (ASP) fee acquisition and average daily antimicrobial costs. Predicted average daily antimicrobial costs in the propensity score-matched cohort (*n* = 926) from Tweedie generalized linear mixed models illustrating the interaction between hospital size and ASP fee acquisition. Abbreviations: ASP, antimicrobial stewardship program; JPY, Japanese Yen

This modifying effect also extended to average daily antimicrobial costs (Fig. 4). Hospital size significantly modified the association between ASP fee acquisition and average daily costs of antipseudomonal drugs (P for interaction < 0.01). Further breakdown by route of administration revealed a more detailed pattern of prescribing shifts, with distinct effects observed for injectable (Supplementary Fig. 1) and oral (Supplementary Fig. 2) antimicrobials. Hospital size significantly modified the association between ASP fee acquisition and costs for both injectable antipseudomonal drugs (P for interaction < 0.01) and other injectable antimicrobials (P for interaction = 0.03). Specifically, in large hospitals, ASP fee acquisition was associated with substantial reduction in antipseudomonal injectable costs (RR 0.46, 95% CI 0.30–0.71), alongside a trend toward higher costs for other injectable antimicrobials (RR 2.35, 95% CI 0.99–5.59).

### Supplementary analysis

Sensitivity analysis examining antimicrobial use and costs over the entire hospitalization period supported the primary findings. However, it also revealed stronger and more significant impact of ASPs on cost reduction in large hospitals (Supplementary Table 5; Supplementary Figs. 3 and 4). Regarding therapy duration, the results were consistent with the primary analysis, showing that hospital size did not significantly modify the association between ASP fee acquisition and duration for any major category. However, for antimicrobial costs, effect modification by hospital size was more pronounced. In the analysis of the entire hospitalization period, the interaction for total antimicrobial costs became highly significant (P for interaction < 0.01), driven by significant interaction for total injectable antimicrobial costs (P for interaction < 0.01). The interaction for antipseudomonal drugs also became more significant (P for interaction < 0.01). Specifically, in large hospitals, ASP acquisition was associated with significant cost reductions for total antimicrobials (RR 0.52, 95% CI 0.32–0.86) and antipseudomonal drugs (RR 0.31, 95% CI 0.19–0.52).

## Discussion

This nationwide retrospective cohort study using a representative Japanese hospital database found that although patients with MRSA BSIs in large hospitals had similar clinical outcomes to those in non-large hospitals, they incurred significantly higher average daily antimicrobial costs after propensity score matching. Notably, the analysis revealed that hospital size substantially modified the impact of a facility’s ASP, as indicated by acquisition of the national stewardship fee. In non-large hospitals, ASP acquisition was associated with significantly shorter time to discharge. In contrast, in large hospitals, the primary impact of ASPs was on prescribing patterns, characterized by significant cost reductions for certain broad-spectrum agents (e.g., antipseudomonal drugs) without significant effect on time to discharge. These findings highlight that the context-dependent nature of ASP effectiveness is not a limitation but rather indicates that ASPs can serve as flexible and powerful tools for addressing unique clinical challenges inherent in different institutional settings.

Our baseline findings regarding antimicrobial costs are consistent with previous research indicating higher rates of broad-spectrum antimicrobial use in larger tertiary care hospitals [32, 33]. Notably, even before propensity score matching, patients in large hospitals presented with more complex backgrounds, such as higher prevalence of malignancy. This reflects the role of large hospitals in managing severely ill patients. The higher average daily costs for antipseudomonal and antifungal drugs in large hospitals even after careful propensity score matching may reflect institutional differences in prescribing culture, potentially including more aggressive or prolonged empirical therapy [34, 35]. 

In this context, the comparable clinical outcomes suggest that this higher expenditure may not translate into better results for this matched patient population, raising the possibility of less optimized prescribing. Our findings on the effect of ASPs support this interpretation. The primary impact of ASPs in large hospitals was on prescribing patterns rather than on length of stay. This suggests that in resource-rich settings, where fundamental clinical indicators such as length of stay may already be managed to high standards, ASPs instead address the more specialized challenge of optimizing pharmacotherapy. Therefore, the observed reduction in costs for specific broad-spectrum agents should be interpreted not only as a cost-saving measure but also as evidence of prescribing refinement that contributes to the quality of medical care.

This prescribing refinement is best characterized as a qualitative shift in antimicrobial use. The significant cost reductions for broad-spectrum agents (e.g., antipseudomonal injectables) without corresponding change in overall treatment duration are characteristic of de-escalation—a cornerstone of advanced antimicrobial stewardship [36]. In patients with severe infection, it is common to initiate empirical therapy with broad-spectrum agents and subsequently switch to narrower-spectrum agents as the patient’s condition and culture results become clear. The reduction in costs for broad-spectrum antipseudomonal agents, along with a trend toward higher costs for other injectable antimicrobials, likely indicates such movement toward more appropriate, targeted antimicrobial use. This suggests that in resource-rich settings, ASPs may focus on more sophisticated interventions such as de-escalation, a practice that has been shown to be safe and effective for enhancing rational use of antibiotics [37, 38]. 

In clear contrast to the prescribing refinements observed in large hospitals, ASPs in non-large hospitals appeared to function primarily as tools to optimize clinical efficiency. This profound differential effect highlights that challenges and opportunities for antimicrobial stewardship are highly context-dependent. The strong and statistically significant association with shorter time to discharge represents an extremely valuable outcome, signifying improvement in the clinical process that benefits both patients and hospital management. The absence of corresponding reduction in drug costs is consistent with our baseline finding that these facilities have lower expenditures on expensive broad-spectrum antimicrobials, which provide limited room for cost-saving interventions. It is instead plausible that the ability to implement a nationally incentivized ASP itself reflects an established efficient medical care system. This underlying organizational strength, facilitated by the ASP, may drive the overall improvement in therapeutic processes, leading to shorter length of stay. Our results are consistent with those of previous studies in community and smaller hospital settings [39, 40] that have also demonstrated that ASP implementation can lead to significant reductions in both therapy duration and length of stay.

In addition to the distinct effects on clinical efficiency and prescribing patterns, a common trend observed in both hospital types is worth noting. Although not statistically significant, the trend toward increased duration of oral antimicrobial use may indicate another positive ASP outcome: promotion of intravenous-to-oral switch therapy. This practice is a key stewardship intervention known to be safe and effective, reducing risks associated with intravenous lines and lowering healthcare costs without compromising clinical outcomes.

Our findings should be interpreted within the context of the propensity score-matched population (*n* = 926). This matching approach balanced patient-level characteristics between hospital size groups, allowing us to isolate effects of institutional context from patient case-mix differences. Although the matching necessarily limited the representativeness within each hospital-size stratum, the primary finding—ASPs have qualitatively different impacts in large versus non-large hospitals—remained valid and robust. The interaction term tested whether the ASP-outcome association differed by hospital size, which was validly estimated regardless of within-stratum representativeness. Therefore, the stratum-specific effects reported in this study apply to patients with comparable baseline characteristics across hospital types, and the demonstration of effect modification provides important evidence that institutional context fundamentally shapes how ASPs function.

This study has several limitations inherent in its observational design using a nationwide administrative claims database. First, as in all observational studies, potential for residual confounding from unmeasured variables remains despite propensity score matching. Administrative data, collected primarily for reimbursement, inherently lack granular clinical details that could influence outcomes. These include the adequacy of source control and microbiological clearance, as well as antimicrobial susceptibility patterns and minimum inhibitory concentrations for MRSA isolates. The variation in MRSA susceptibility profiles could influence treatment selection, duration, and outcomes. However, national surveillance data indicate that vancomycin resistance in MRSA remains extremely rare in Japan [41], and the consistent treatment patterns observed across both hospital types suggest that major resistance-related treatment barriers were not systematically present during the study period.

Second, the ASP definition relied on a structural reimbursement marker, and ASP fee acquisition itself was not directly adjusted through matching. While this is a standard proxy in large-scale database research, this code identifies presence of a fee-eligible structure, not the process or quality of its activities (e.g., program maturity and implementation intensity). The ASP fee represents a facility-level structural indicator rather than a randomized intervention, and residual confounding by unmeasured facility-level factors cannot be fully excluded. Therefore, our findings should be interpreted as evidence of differential associations in real-world practice rather than definitive evidence of causal effects. Nevertheless, the observed effects reflect the impact of having this formal structure in real-world settings, and consistency with prior literature and biologically plausible mechanisms support the validity of our conclusions.

Finally, patients transferred between hospitals were excluded. This was methodologically necessary to analyze the complete episode of care and accurately assess outcomes like time to discharge but may limit generalizability to large tertiary referral centers. Despite these limitations, our study provides valuable insights into real-world clinical practice. Strengths include nationwide scope, robust matching methodology, and novel examination of effect modification. Our results support that a “one-size-fits-all” approach to antimicrobial stewardship is likely suboptimal. This has important policy implications, as it suggests that stewardship incentives and performance metrics should be tailored to specific institutional settings. For small hospitals, metrics focused on clinical efficiency, such as length of stay, may be most relevant. Meanwhile, for large institutions, metrics might focus on appropriate use of targeted high-cost antimicrobials. Future research should focus on identifying specific components of ASPs that drive these differential outcomes to further optimize antimicrobial use and patient care across the entire healthcare system.

## Conclusions

Hospital size significantly modified the association between ASP fee acquisition and outcomes among patients with MRSA BSI. In non-large hospitals, ASP fee acquisition was associated with shorter time to discharge, suggesting improved clinical efficiency. In large hospitals, ASP fee acquisition was primarily associated with changes in prescribing patterns, such as de-escalation of broad-spectrum agents. These differential associations highlight the need for stewardship evaluation and policy to be tailored to institutional characteristics.

## Supplementary Information

Below is the link to the electronic supplementary material.

Supplementary Material 1

## Data Availability

The data that support the findings of this study are available from JMDC Inc. but restrictions apply to the availability of these data, which were used under license for the current study and so are not publicly available. Data are however available from the authors upon reasonable request and with permission of JMDC Inc. The R script used for all statistical analyses is available in the supplementary materials.

## Abbreviations

ASP: Antimicrobial stewardship program
BSI: Bloodstream infection
CCI: Charlson Comorbidity Index
CI: Confidence interval
DPC: Diagnosis Procedure Combination
GLMM: Generalized linear mixed model
HR: Hazard ratio
JMDC: Japan Medical Data Center
JPY: Japanese Yen
MRSA: Methicillin-resistant Staphylococcus aureus
OR: Odds ratio
RR: Rate ratio
SMD: Standardized mean difference
SOFA: Sequential Organ Failure Assessment
