# Supplementary material for: Modifying effect of hospital size on the impact of antimicrobial stewardship programs for methicillin-resistant Staphylococcus aureus bloodstream infections: a nationwide claims database analysis
Source: J Pharm Health Care Sci. 2026 Feb 26;12:39. doi: 10.1186/s40780-026-00558-2 (PMC13041119; doi:10.1186/s40780-026-00558-2)
Supplement: Supplementary file 1 — Supplementary Material 1 [file 40780_2026_558_MOESM1_ESM.docx]

**Supplementary Materials**

**Supplementary Table 1. Definitions of study cohort criteria, key variables, outcomes, and covariates**

| Category | Variable | Definition and Logic |
| --- | --- | --- |
| Inclusion Criteria | MRSA Sepsis | Patients with an inpatient diagnosis corresponding to the Japanese Standard Disease Code 8830124, which maps to the ICD-10 code A41.0 (Sepsis due to Staphylococcus aureus). |
|  | Facility with Infection Prevention Fee 1 | Hospitalization at a facility billing for Infection Prevention Fee 1 (Japanese Standardized Procedure Code 190188810). This fee serves as a structural marker for a dedicated infection control team and is a prerequisite for claiming the Antimicrobial Stewardship Fee. |
| Exclusion Criteria | Missing claims data | Patients with incomplete hospitalization records owing to the JMDC data collection structure. The JMDC database aggregates claims on a monthly basis directly from contracted hospitals. When a patient's hospitalization spanned multiple months and the facility did not submit complete claims data for all relevant months, the hospitalization episode contained missing data. Such patients were excluded to ensure the accurate assessment of the complete episode of care. |
|  | Transfer from another hospital | Patients with an admission route code of 4 in the Form 1 file. |
|  | Transfer to another hospital | Patients with a discharge destination code of 4 in the Form 1 file. |
|  | No anti-MRSA drug use | Patients with no claims records for any of the anti-MRSA agents listed in Supplementary Table 3. |
|  | Missing SOFA score data | Patients with any missing components of the SOFA score in the Form 1 SOFA file. |
| Exposure & Effect Modifier | Hospital Size | Classified based on the number of beds in the claims file:   • Large hospital: 500+   • Non-large hospital: 20-99, 100-199, 200-299, or 300-499 |
|  | Antimicrobial Stewardship Program (ASP) | Hospitalization at a facility billing the Japanese Standardized Procedure Code 190206870 (Antimicrobial Stewardship Fee). |
| Outcomes | MRSA BSI Onset (Day 0) | The date of the first SOFA score measurement recorded in the Form 1 SOFA file. |
|  | 30-day Mortality | Discharge outcome codes of 6 (Death due to primary disease) or 7 (Death due to other causes) in the Form 1 file. |
|  | 30-day Unplanned Readmission | Unplanned readmissions were identified based on the readmission classification in the Form 1 file of the database, by excluding admissions explicitly coded as “planned.” |
| Covariates | Care facility resident | Patients with an admission route code of 5 (Admitted from a long-term care/welfare facility) in the Form 1 file. |
|  | Infective Endocarditis | Patients with any of the following Japanese Standard Disease Codes, which all map to ICD-10 code I33.0: 4210009, 8830116, 8832314, 8830176. |
|  | Surgery | Patients with at least one record in the Form 1 Surgery file for their admission ID. |
|  | Central venous catheter placement | Patients with claims for any of the following Japanese Standardized Procedure Codes: 130004670, 130011570, 130011610, 130011810. |
|  | Dialysis | Patients with claims for any of the following Japanese Standardized Procedure Codes: 140057810-140058610 (Hemodialysis for chronic maintenance), 140007710 (Hemodialysis, other), 140029850 (Continuous hemofiltration), or any related add-on codes. |

This table provides operational definitions for all variables used in this study based on the Japan Medical Data Center (JMDC) claims database. To ensure transparency and reproducibility in accordance with the REporting of studies Conducted using Observational Routinely-collected health Data (RECORD) guidelines, we defined each variable using specific Japanese Standard Disease Codes, Japanese Standardized Procedure Codes, or codes from the JMDC Form 1 file. These codes serve as objective and reproducible criteria for identifying the study cohort, exposures, outcomes, and covariates.

**Supplementary Table 2. Charlson Comorbidity Index definitions based on the Quan et al. ICD-10 algorithm**

| Comorbidity | ICD-10 Codes |
| --- | --- |
| Myocardial Infarction | I21.*, I22.*, I25.2 |
| Congestive Heart Failure | I09.9, I11.0, I13.0, I13.2, I25.5, I42.0, I42.5-I42.9, I43.*, I50.*, P29.0 |
| Peripheral Vascular Disease | I70.*, I71.*, I73.1, I73.8, I73.9, I77.1, I79.0, I79.2, K55.1, K55.8, K55.9, Z95.8, Z95.9 |
| Cerebrovascular Disease | G45.*, G46.*, I60.*-I69.*, H34.0 |
| Dementia | F00.*-F03.*, F05.1, G30.*, G31.1 |
| Chronic Pulmonary Disease | I27.8, I27.9, J40.*-J47.*, J60.*-J67.*, J68.4, J70.1, J70.3 |
| Rheumatic Disease | M05.*, M06.*, M31.5, M32.*-M34.*, M35.1, M35.3, M36.0 |
| Peptic Ulcer Disease | K25.*-K28.* |
| Mild Liver Disease | B18.*, K70.0-K70.3, K70.9, K71.3-K71.5, K71.7, K73.*, K74.*, K76.0, K76.2-K76.4, K76.8, K76.9, Z94.4 |
| Diabetes without complication | E10.0, E10.1, E10.6, E10.8, E10.9, E11.0, E11.1, E11.6, E11.8, E11.9, E12.0, E12.1, E12.6, E12.8, E12.9, E13.0, E13.1, E13.6, E13.8, E13.9, E14.0, E14.1, E14.6, E14.8, E14.9 |
| Diabetes with complication | E10.2-E10.5, E10.7, E11.2-E11.5, E11.7, E12.2-E12.5, E12.7, E13.2-E13.5, E13.7, E14.2-E14.5, E14.7 |
| Hemiplegia or Paraplegia | G04.1, G11.4, G80.1, G80.2, G81.*, G82.*, G83.0-G83.4, G83.9 |
| Renal Disease | I12.0, I13.1, N03.2-N03.7, N05.2-N05.7, N18.*, N19.*, N25.0, Z49.0-Z49.2, Z94.0, Z99.2 |
| Malignancy | C00.*-C26.*, C30.*-C34.*, C37.*-C41.*, C43.*, C45.*-C58.*, C60.*-C76.*, C81.*-C85.*, C88.*, C90.*-C97.* |
| Moderate/Severe Liver Disease | I85.0, I85.9, I86.4, I98.2, K70.4, K71.1, K72.1, K72.9, K76.5-K76.7 |
| Metastatic Solid Tumor | C77.*-C80.* |
| AIDS/HIV | B20.*, B21.*, B22.*, B24.* |

This table details the International Classification of Diseases, 10th Revision (ICD-10) codes used to define comorbidities comprising the Charlson Comorbidity Index (CCI) based on the validated algorithm by Quan et al. (2005). Standardized measurement of these comorbidities was essential for risk adjustment performed via propensity score matching in this study.

**Supplementary Table 3. Antimicrobial agent classification**

| Class | Subclass | Agent | ATC Code |
| --- | --- | --- | --- |
| Anti-MRSA Agents | Glycopeptide | Teicoplanin | J01XA02 |
|  | Glycopeptide | Vancomycin | J01XA01 |
|  | Lipopeptide | Daptomycin | J01XX09 |
|  | Oxazolidinone | Linezolid | J01XX08 |
|  | Oxazolidinone | Tedizolid | J01XX11 |
|  | Aminoglycoside | Arbekacin | J01GB12 |
| Anti-pseudomonal Agents | Penicillins | Piperacillin | J01CA12 |
|  | Penicillins (combination) | Piperacillin/Tazobactam | J01CR05 |
|  | Cephalosporins (3rd Gen) | Cefoperazone | J01DD12 |
|  | Cephalosporins (3rd Gen) | Ceftazidime | J01DD02 |
|  | Cephalosporins (3rd Gen, combination) | Cefoperazone/Sulbactam | J01DD62 |
|  | Cephalosporins (3rd Gen, combination) | Ceftazidime/Avibactam | J01DD52 |
|  | Cephalosporins (4th Gen) | Cefepime | J01DE01 |
|  | Cephalosporins (4th Gen) | Cefozopran | J01DE03 |
|  | Cephalosporins (4th Gen) | Cefpirome | J01DE02 |
|  | Cephalosporins (combination) | Ceftolozane/Tazobactam | J01DI54 |
|  | Siderophore Cephalosporins | Cefiderocol | J01DI04 |
|  | Carbapenems | Doripenem | J01DH04 |
|  | Carbapenems | Biapenem | J01DH05 |
|  | Carbapenems | Meropenem | J01DH02 |
|  | Carbapenems (combination) | Imipenem/Cilastatin | J01DH51 |
|  | Carbapenems (combination) | Imipenem/Cilastatin/Relebactam | J01DH56 |
|  | Carbapenems (combination) | Panipenem/Betamipron | J01DH55 |
|  | Monobactams | Aztreonam | J01DF01 |
|  | Fluoroquinolones | Ciprofloxacin | J01MA02 |
|  | Fluoroquinolones | Pazufloxacin | J01MA18 |
|  | Fluoroquinolones | Levofloxacin | J01MA12 |
|  | Fluoroquinolones | Ofloxacin | J01MA01 |
|  | Fluoroquinolones | Sitafloxacin | J01MA21 |
|  | Fluoroquinolones | Tosufloxacin | J01MA22 |
|  | Fluoroquinolones | Norfloxacin | J01MA06 |
|  | Fluoroquinolones | Prulifloxacin | J01MA17 |
|  | Fluoroquinolones | Lomefloxacin | J01MA07 |
|  | Quinolones | Pipemidic acid | J01MB04 |
|  | Aminoglycosides | Amikacin | J01GB06 |
|  | Aminoglycosides | Isepamicin | J01GB11 |
|  | Aminoglycosides | Gentamicin | J01GB03 |
|  | Aminoglycosides | Dibekacin | J01GB09 |
|  | Aminoglycosides | Tobramycin | J01GB01 |
|  | Polymyxins | Colistin | J01XB01 |
| Other Antimicrobials | Penicillins | Benzylpenicillin | J01CE01 |
|  | Penicillins | Benzathine benzylpenicillin | J01CE08 |
|  | Penicillins | Aspoxicillin | J01CA19 |
|  | Penicillins | Ampicillin | J01CA01 |
|  | Penicillins | Amoxicillin | J01CA04 |
|  | Penicillins | Bacampicillin | J01CA06 |
|  | Penicillins | Pivmecillinam | J01CA08 |
|  | Penicillins (combination) | Ampicillin/Cloxacillin | J01CR50 |
|  | Penicillins (combination) | Ampicillin/Sulbactam | J01CR01 |
|  | Penicillins (combination) | Sultamicillin | J01CR04 |
|  | Penicillins (combination) | Amoxicillin/Clavulanic acid | J01CR02 |
|  | Cephalosporins (1st Gen) | Cefazolin | J01DB04 |
|  | Cephalosporins (1st Gen) | Cefalotin | J01DB03 |
|  | Cephalosporins (1st Gen) | Cefalexin | J01DB01 |
|  | Cephalosporins (1st Gen) | Cefroxadine | J01DB11 |
|  | Cephalosporins (2nd Gen) | Cefotiam | J01DC07 |
|  | Cephalosporins (2nd Gen) | Cefminox | J01DC12 |
|  | Cephalosporins (2nd Gen) | Cefmetazole | J01DC09 |
|  | Cephalosporins (2nd Gen) | Cefaclor | J01DC04 |
|  | Cephalosporins (2nd Gen) | Cefuroxime | J01DC02 |
|  | Cephalosporins (3rd Gen) | Cefotaxime | J01DD01 |
|  | Cephalosporins (3rd Gen) | Ceftriaxone | J01DD04 |
|  | Cephalosporins (3rd Gen) | Cefmenoxime | J01DD05 |
|  | Cephalosporins (3rd Gen) | Cefodizime | J01DD09 |
|  | Cephalosporins (3rd Gen) | Cefixime | J01DD08 |
|  | Cephalosporins (3rd Gen) | Cefcapene | J01DD17 |
|  | Cephalosporins (3rd Gen) | Cefditoren | J01DD16 |
|  | Cephalosporins (3rd Gen) | Cefdinir | J01DD15 |
|  | Cephalosporins (3rd Gen) | Ceftibuten | J01DD14 |
|  | Cephalosporins (3rd Gen) | Cefteram | J01DD18 |
|  | Cephalosporins (3rd Gen) | Cefpodoxime | J01DD13 |
|  | Oxacephems | Flomoxef | J01DC14 |
|  | Oxacephems | Latamoxef | J01DD06 |
|  | Carbapenems | Tebipenem | J01DH06 |
|  | Carbapenems | Faropenem | J01DI03 |
|  | Fluoroquinolones | Lascufloxacin | J01MA25 |
|  | Fluoroquinolones | Garenoxacin | J01MA19 |
|  | Fluoroquinolones | Moxifloxacin | J01MA14 |
|  | Quinolones | Cinoxacin | J01MB06 |
|  | Quinolones | Nalidixic acid | J01MB02 |
|  | Macrolides | Azithromycin | J01FA10 |
|  | Macrolides | Erythromycin | J01FA01 |
|  | Macrolides | Clarithromycin | J01FA09 |
|  | Macrolides | Josamycin | J01FA07 |
|  | Macrolides | Spiramycin | J01FA02 |
|  | Macrolides | Roxithromycin | J01FA06 |
|  | Macrolides | Rokitamycin | J01FA12 |
|  | Lincosamides | Clindamycin | J01FF01 |
|  | Lincosamides | Lincomycin | J01FF02 |
|  | Aminoglycosides | Sulfamethoxazole/Trimethoprim | J01EE01 |
|  | Aminoglycosides | Kanamycin | J01GB04 |
|  | Aminoglycosides | Spectinomycin | J01XX04 |
|  | Aminoglycosides | Ribostamycin | J01GB10 |
|  | Aminoglycosides | Streptomycin | J01GA01 |
|  | Tetracyclines | Minocycline | J01AA08 |
|  | Tetracyclines | Tetracycline | J01AA07 |
|  | Tetracyclines | Demeclocycline | J01AA01 |
|  | Tetracyclines | Doxycycline | J01AA02 |
|  | Glycylcyclines | Tigecycline | J01AA12 |
|  | Streptogramins | Quinupristin/Dalfopristin | J01FG02 |
|  | Amphenicols | Chloramphenicol | J01BA01 |
|  | Sulfonamides | Sulfadimethoxine | J01ED01 |
|  | Nitroimidazoles | Metronidazole | J01XD01, P01AB01 |
|  | Nitroimidazoles | Tinidazole | P01AB02 |
|  | Others | Fosfomycin | J01XX01 |
|  | Others (for intestinal infections) | Fidaxomicin | A07AA12 |
|  | Others (for intestinal infections) | Kanamycin | A07AA08 |
|  | Others (for intestinal infections) | Vancomycin | A07AA09 |
|  | Others (for intestinal infections) | Colistin | A07AA10 |
|  | Others (for intestinal infections) | Polymyxin B | A07AA05 |
|  | Others (for intestinal infections) | Rifaximin | A07AA11 |
| Antifungal Agents | Polyenes | Amphotericin B | J02AA01 |
|  | Polyenes | Liposomal amphotericin B | J02AA01 |
|  | Azoles | Miconazole | J02AB01 |
|  | Azoles | Isavuconazole | J02AC05 |
|  | Azoles | Itraconazole | J02AC02 |
|  | Azoles | Fluconazole | J02AC01 |
|  | Azoles | Posaconazole | J02AC04 |
|  | Azoles | Fosfluconazole | J02AC01 |
|  | Azoles | Voriconazole | J02AC03 |
|  | Echinocandins | Caspofungin | J02AX04 |
|  | Echinocandins | Micafungin | J02AX05 |
|  | Others | Flucytosine | J02AX01 |
| Antiviral Agents | Anti-herpesvirus | Aciclovir | J05AB01 |
|  | Anti-herpesvirus | Ganciclovir | J05AB06 |
|  | Anti-herpesvirus | Vidarabine | J05AB03 |
|  | Anti-herpesvirus | Famciclovir | J05AB09 |
|  | Anti-herpesvirus | Valaciclovir | J05AB11 |
|  | Anti-herpesvirus | Valganciclovir | J05AB14 |
|  | Anti-herpesvirus | Amenamevir | J05AX26 |
|  | Anti-cytomegalovirus | Foscarnet | J05AD01 |
|  | Anti-cytomegalovirus | Letermovir | J05AX18 |
|  | Anti-cytomegalovirus | Maribavir | J05AX10 |
|  | Anti-influenza | Peramivir | J05AH03 |
|  | Anti-influenza | Baloxavir marboxil | J05AX25 |
|  | Anti-influenza | Oseltamivir | J05AH02 |
|  | Anti-HCV | Asunaprevir | J05AP06 |
|  | Anti-HCV | Daclatasvir | J05AP07 |
|  | Anti-HCV | Daclatasvir, asunaprevir and beclabuvir | J05AP58 |
|  | Anti-HCV | Elbasvir | J05AP10 |
|  | Anti-HCV | Glecaprevir and pibrentasvir | J05AP57 |
|  | Anti-HCV | Grazoprevir | J05AP11 |
|  | Anti-HCV | Simeprevir | J05AP05 |
|  | Anti-HCV | Sofosbuvir | J05AP08 |
|  | Anti-HCV | Sofosbuvir and ledipasvir | J05AP51 |
|  | Anti-HCV | Sofosbuvir and velpatasvir | J05AP55 |
|  | Anti-HCV | Telaprevir | J05AP02 |
|  | Anti-HCV | Vaniprevir | J05AEXB |
|  | Anti-HIV (NRTI) | Abacavir | J05AF06 |
|  | Anti-HIV (NRTI) | Didanosine | J05AF02 |
|  | Anti-HIV (NRTI) | Emtricitabine | J05AF09 |
|  | Anti-HIV (NRTI) | Lamivudine | J05AF05 |
|  | Anti-HIV (NRTI) | Stavudine | J05AF04 |
|  | Anti-HIV (NRTI) | Zidovudine | J05AF01 |
|  | Anti-HIV (NtRTI) | Adefovir dipivoxil | J05AF08 |
|  | Anti-HIV (NtRTI) | Tenofovir alafenamide | J05AF13 |
|  | Anti-HIV (NtRTI) | Tenofovir disoproxil | J05AF07 |
|  | Anti-HIV (NNRTI) | Delavirdine | J05AG02 |
|  | Anti-HIV (NNRTI) | Doravirine | J05AG06 |
|  | Anti-HIV (NNRTI) | Efavirenz | J05AG03 |
|  | Anti-HIV (NNRTI) | Etravirine | J05AG04 |
|  | Anti-HIV (NNRTI) | Nevirapine | J05AG01 |
|  | Anti-HIV (NNRTI) | Rilpivirine | J05AG05 |
|  | Anti-HIV (PI) | Atazanavir | J05AE08 |
|  | Anti-HIV (PI) | Darunavir | J05AE10 |
|  | Anti-HIV (PI) | Fosamprenavir | J05AE07 |
|  | Anti-HIV (PI) | Indinavir | J05AE02 |
|  | Anti-HIV (PI) | Nelfinavir | J05AE04 |
|  | Anti-HIV (PI) | Ritonavir | J05AE03 |
|  | Anti-HIV (PI) | Saquinavir | J05AE01 |
|  | Anti-HIV (INSTI) | Cabotegravir | J05AJ04 |
|  | Anti-HIV (INSTI) | Dolutegravir | J05AJ03 |
|  | Anti-HIV (INSTI) | Raltegravir | J05AJ01 |
|  | Anti-HIV (CCR5 antagonist) | Maraviroc | J05AX09 |
|  | Anti-HIV (Capsid inhibitor) | Lenacapavir | J05AX31 |
|  | Anti-HIV (Combinations) | Darunavir and cobicistat | J05AR14 |
|  | Anti-HIV (Combinations) | Dolutegravir and rilpivirine | J05AR21 |
|  | Anti-HIV (Combinations) | Emtricitabine and tenofovir alafenamide | J05AR17 |
|  | Anti-HIV (Combinations) | Emtricitabine, tenofovir alafenamide and bictegravir | J05AR20 |
|  | Anti-HIV (Combinations) | Emtricitabine, tenofovir alafenamide and rilpivirine | J05AR19 |
|  | Anti-HIV (Combinations) | Emtricitabine, tenofovir alafenamide, darunavir and cobicistat | J05AR22 |
|  | Anti-HIV (Combinations) | Emtricitabine, tenofovir alafenamide, elvitegravir and cobicistat | J05AR18 |
|  | Anti-HIV (Combinations) | Emtricitabine, tenofovir disoproxil and rilpivirine | J05AR08 |
|  | Anti-HIV (Combinations) | Emtricitabine, tenofovir disoproxil, elvitegravir and cobicistat | J05AR09 |
|  | Anti-HIV (Combinations) | Lamivudine and abacavir | J05AR02 |
|  | Anti-HIV (Combinations) | Lamivudine and dolutegravir | J05AR25 |
|  | Anti-HIV (Combinations) | Lamivudine, abacavir and dolutegravir | J05AR13 |
|  | Anti-HIV (Combinations) | Lopinavir and ritonavir | J05AR10 |
|  | Anti-HIV (Combinations) | Tenofovir disoproxil and emtricitabine | J05AR03 |
|  | Anti-HIV (Combinations) | Zidovudine and lamivudine | J05AR01 |
|  | Anti-HBV | Entecavir | J05AF10 |
|  | COVID-19 | Remdesivir | J05AB16 |
|  | COVID-19 | Ensitrelvir | J05AE16 |
|  | COVID-19 | Favipiravir | J05AX27 |
|  | COVID-19 | Molnupiravir | J05AB18 |
|  | COVID-19 | Nirmatrelvir and ritonavir | J05AE30 |
|  | Others | Ribavirin | J05AP01 |
|  | Others | Ombitasvir, paritaprevir and ritonavir | J05AP53 |

This table provides a comprehensive classification of all antimicrobial, antifungal, and antiviral agents included in the analysis, categorized according to the Anatomical Therapeutic Chemical (ATC) classification system. The agents are organized into the clinically relevant groups used in the outcome analysis.

**Supplementary Table 4. Comparison of average daily injectable and oral antimicrobial costs from onset to discharge in propensity score-matched cohorts**

| Outcome | Non-large hospitals (n=463) | Large hospitals (n=463) | Effect measure (95% CI) | P-value |
| --- | --- | --- | --- | --- |
| Injectable antimicrobial costs (JPY) |  |  |  |  |
| Total injectable antimicrobial costs | 1,662 [832–3,637] | 2,026 [998–4,500] | RR 1.33 [1.17–1.51] | < 0.01 |
| Injectable anti-MRSA drug costs | 649 [343–1,697] | 843 [345–1,870] | RR 1.12 [0.96–1.29] | 0.14 |
| Injectable anti-pseudomonal drug costs | 358 [0–1,061] | 359 [0–1,200] | RR 1.18 [1.00–1.39] | 0.05 |
| Injectable other antimicrobial costs | 13 [0–159] | 30 [0–259] | RR 1.31 [1.05–1.65] | 0.02 |
| Injectable antifungal drug costs | 0 [0–0] | 0 [0–0] | RR 2.43 [1.51–3.89] | < 0.01 |
| Injectable antiviral drug costs | 0 [0–0] | 0 [0–0] | RR 1.62 [0.62–4.22] | 0.32 |
| Oral antimicrobial costs (JPY) |  |  |  |  |
| Total oral antimicrobial costs | 0 [0–29] | 0 [0–63] | RR 1.42 [1.03–1.96] | 0.03 |
| Oral anti-MRSA drug costs | 0 [0–0] | 0 [0–0] | RR 1.08 [0.46–2.54] | 0.87 |
| Oral anti-pseudomonal drug costs | 0 [0–0] | 0 [0–0] | RR 1.03 [0.69–1.54] | 0.88 |
| Oral other antimicrobial costs | 0 [0–8] | 0 [0–14] | RR 1.06 [0.75–1.51] | 0.72 |
| Oral antifungal drug costs | 0 [0–0] | 0 [0–0] | RR 1.70 [1.01–2.87] | 0.05 |
| Oral antiviral drug costs | 0 [0–0] | 0 [0–0] | RR 2.28 [1.05–4.96] | 0.04 |

Data are presented as median [interquartile range].

Abbreviations: CI, confidence interval; JPY, Japanese Yen; RR, rate ratio.

We estimated RRs for average daily costs using Tweedie generalized linear mixed models to account for zero-inflated and right-skewed cost distributions.

A statistically significant P-value for cost categories with median of 0 indicates significant difference in the frequency or magnitude of non-zero costs between groups.

**Supplementary Table 5.** Sensitivity analysis: comparison of antimicrobial therapy duration and average daily costs during the entire hospitalization period in propensity score-matched cohorts

| Outcome | Non-large hospitals (n=463) | Large hospitals (n=463) | Effect measure (95% CI) | P-value |
| --- | --- | --- | --- | --- |
| Duration of antimicrobial therapy (days) |  |  |  |  |
| Total antimicrobial treatment | 23 [14–39] | 23 [14–43] | RR 1.01 [0.92–1.11] | 0.87 |
| Anti-MRSA drug treatment | 11 [5–18] | 10 [5–16] | RR 0.96 [0.86–1.06] | 0.43 |
| Antimicrobial costs (JPY) |  |  |  |  |
| Total antimicrobial costs | 1,632 [796–3,3729] | 1,959 [979–4,492] | RR 1.28 [1.13–1.45] | < 0.01 |
| Total injectable antimicrobial costs | 1,518 [756–3,394] | 1,839 [898–3,905] | RR 1.29 [1.14–1.46] | < 0.01 |
| Injectable anti-MRSA drug costs | 549 [262–1,341] | 664 [280–1,461] | RR 1.08 [0.93–1.25] | 0.31 |
| Injectable anti-pseudomonal drug costs | 350 [27–1,013] | 390 [0–1,098] | RR 1.11 [0.95–1.30] | 0.18 |
| Injectable other antimicrobial costs | 45 [0–183] | 53 [0–270] | RR 1.28 [1.05–1.55] | 0.01 |
| Injectable antifungal drug costs | 0 [0–0] | 0 [0–0] | RR 2.50 [1.61–3.88] | < 0.01 |
| Injectable antiviral drug costs | 0 [0–0] | 0 [0–0] | RR 1.90 [0.82–4.41] | 0.13 |
| Total oral antimicrobial costs | 0 [0–33] | 4 [0–61] | RR 1.44 [1.05–1.97] | 0.02 |
| Oral anti-MRSA drug costs | 0 [0–0] | 0 [0–0] | RR 1.00 [0.43–2.30] | 0.99 |
| Oral anti-pseudomonal drug costs | 0 [0–0] | 0 [0–0] | RR 1.16 [0.79–1.70] | 0.45 |
| Oral other antimicrobial costs | 0 [0–10] | 0 [0–16] | RR 1.09 [0.79–1.51] | 0.61 |
| Oral antifungal drug costs | 0 [0–0] | 0 [0–0] | RR 1.74 [1.04–2.90] | 0.04 |
| Oral antiviral drug costs | 0 [0–0] | 0 [0–0] | RR 1.82 [0.86–3.84] | 0.12 |

Data are presented as median [interquartile range].

Abbreviations: CI, confidence interval; JPY, Japanese Yen; RR, rate ratio.

We estimated RRs for antimicrobial duration using negative binomial generalized linear mixed models.

We estimated RRs for average daily costs using Tweedie generalized linear mixed models.

A statistically significant P-value for cost categories with median of 0 indicates significant difference in the frequency or magnitude of non-zero costs between groups.

**
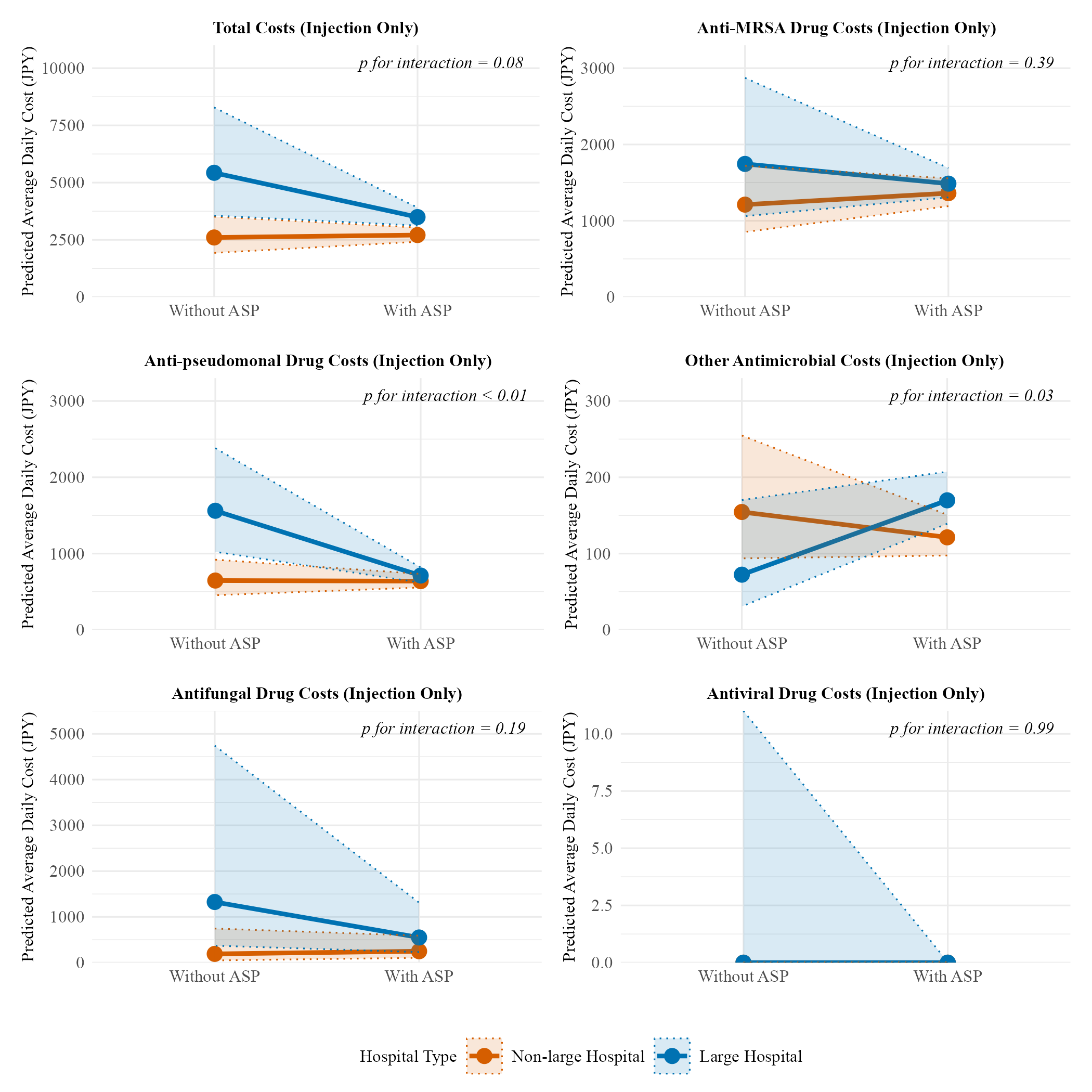
**

**Supplementary Figure 1. Effect modification by hospital size on the association between antimicrobial stewardship program (ASP) fee acquisition and average daily costs for injectable antimicrobials**

Predicted average daily costs for injectable antimicrobials derived from Tweedie generalized linear mixed models illustrating the interaction between hospital size and ASP fee acquisition for different drug classes.

Abbreviations: ASP, antimicrobial stewardship program; JPY, Japanese Yen.


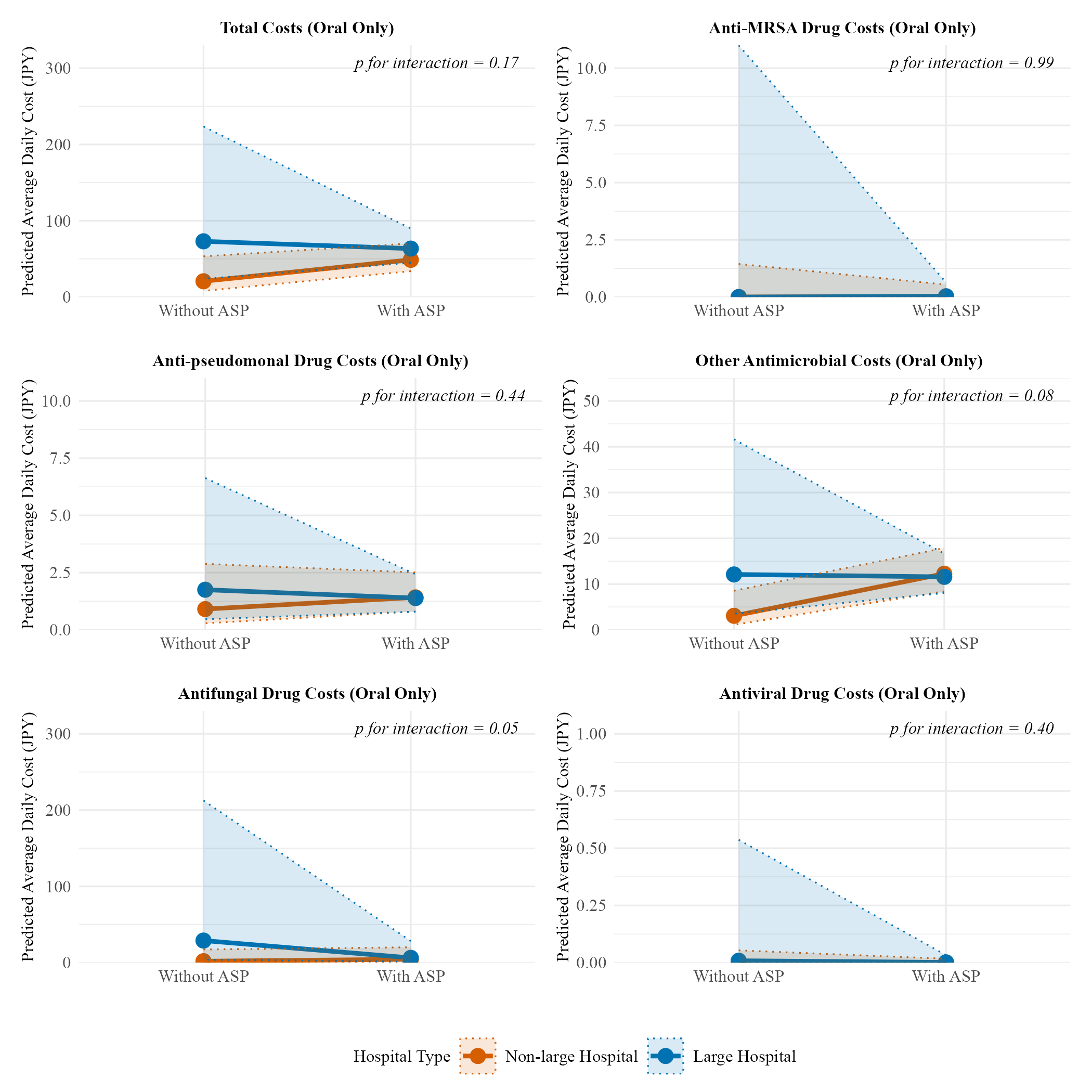


**Supplementary Figure 2. Effect modification by hospital size on the association between antimicrobial stewardship program (ASP) fee acquisition and average daily costs for oral antimicrobials**

Predicted average daily costs for oral antimicrobials derived from Tweedie generalized linear mixed models illustrating the interaction between hospital size and ASP fee acquisition for different drug classes.

Abbreviations: ASP, antimicrobial stewardship program; JPY, Japanese Yen.


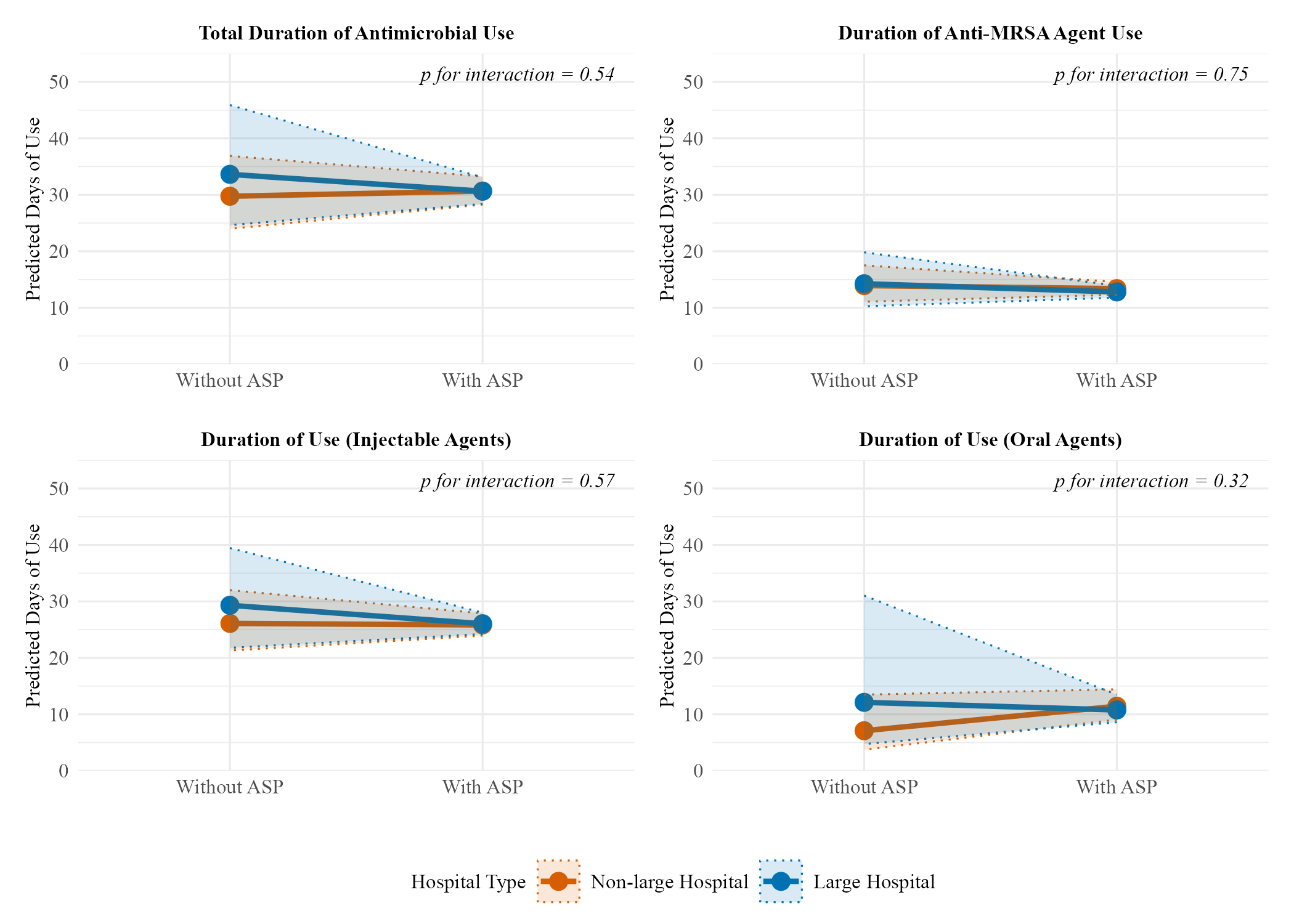


**Supplementary Figure 3. Sensitivity analysis: effect modification by hospital size on the association between ASP fee acquisition and antimicrobial therapy duration during the entire hospitalization period**

Sensitivity analysis showing predicted durations of antimicrobial therapy from negative binomial generalized linear mixed models over the entire hospitalization period.

Abbreviations: ASP, antimicrobial stewardship program.


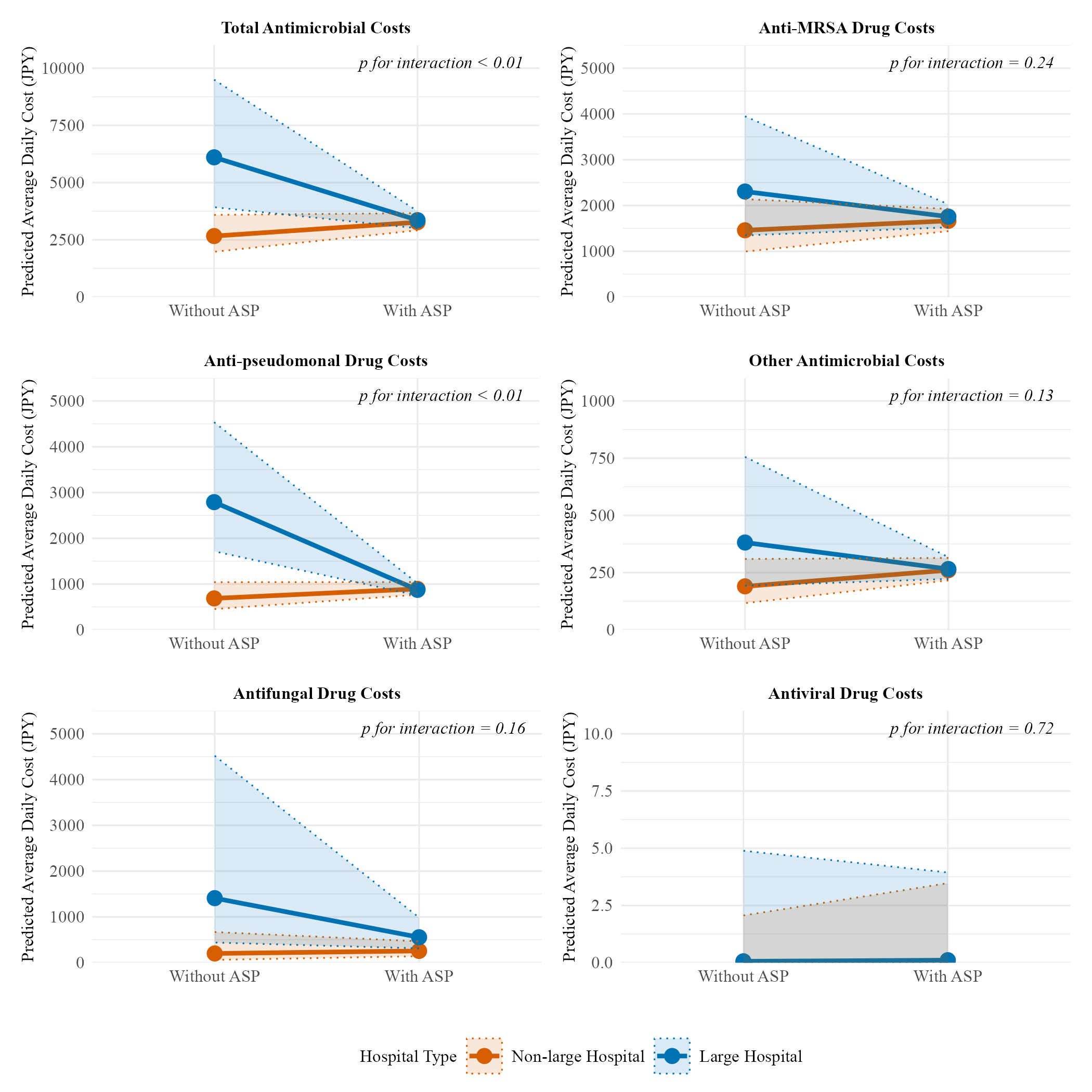


**Supplementary Figure 4. Sensitivity analysis: effect modification by hospital size on the association between ASP fee acquisition and average daily antimicrobial costs during the entire hospitalization period**

Predicted average daily antimicrobial costs during the entire hospitalization period based on Tweedie generalized linear mixed models.

Abbreviations: ASP, antimicrobial stewardship program; JPY, Japanese Yen.

**Appendix S1: R Code for Statistical Analyses**

# --------------------------------------------------------------------------

# R ANALYSIS SCRIPT FOR:

# Modifying Effect of Hospital Size on the Impact of Antimicrobial

# Stewardship Programs for Methicillin-resistant Staphylococcus aureus

# Bloodstream Infections: A Nationwide Claims Database Analysis

#

# Author: Keisuke Sawada, Ryo Inose, Yuichi Muraki.

# Journal: Journal of Pharmaceutical Health Care and Sciences (JPHCS)

# Date of Code Finalization: 2025-12-10

#

# Description:

# This script performs all statistical analyzes presented in the manuscript,

# including baseline comparisons (Table 2 & 3) and effect modification

# analyzes (Figure 2, 3, & 4).

#

# It includes a synthetic data generation process, allowing the code to be

# run and the methodology to be verified without access to the original

# confidential claims data. The synthetic dataset has been enlarged to

# ensure stable model convergence for demonstration purposes.

#

# !! PERFORMANCE NOTE !!

# The generalized linear mixed models (glmmTMB) in SECTION 4 are

# computationally intensive, particularly the Tweedie models.

# Depending on your machine's specifications and the sample size (n_pairs),

# R may appear unresponsive for several seconds to minutes while

# fitting these models. This is expected behavior.

# --------------------------------------------------------------------------

# ==========================================================================

# SECTION 1: SETUP - LOAD LIBRARIES

# ==========================================================================

# --- 1.1: Load Required Libraries ---

# install.packages(c("survival", "glmmTMB", "dplyr"))

library(survival)

library(glmmTMB)

library(dplyr)

# ==========================================================================

# SECTION 2: DATA PREPARATION - SYNTHETIC DATASET

# ==========================================================================

# --- 2.1: Create a More Robust Synthetic Dataset for Reproducibility ---

set.seed(2025) # For reproducibility

n_pairs <- 500 # Increased sample size for model stability

df <- data.frame(

# --- Identifiers and Main Variables ---

matched_pair_id = factor(rep(1:n_pairs, each = 2)),

hospital_size_large = rep(c(0, 1), times = n_pairs), # 0=Non-large, 1=Large

asp_fee_status = rbinom(n_pairs * 2, 1, 0.6),

# --- Clinical Outcomes ---

days_to_discharge = rpois(n_pairs * 2, lambda = 28),

discharged_alive_indicator = rbinom(n_pairs * 2, 1, 0.88),

mortality_30d = rbinom(n_pairs * 2, 1, 0.22),

readmission_30d = rbinom(n_pairs * 2, 1, 0.18),

# --- Duration Outcomes ---

duration_total = rpois(n_pairs * 2, lambda = 21),

duration_anti_mrsa = rpois(n_pairs * 2, lambda = 12),

duration_injectable = rpois(n_pairs * 2, lambda = 15),

duration_oral = rpois(n_pairs * 2, lambda = 7),

# --- Cost Outcomes (Total JPY) ---

cost_total_jpy = rgamma(n_pairs * 2, shape = 5, scale = 1600),

cost_anti_mrsa_jpy = rgamma(n_pairs * 2, shape = 3, scale = 1100),

cost_antipseudomonal_jpy = rgamma(n_pairs * 2, shape = 2, scale = 450) * rbinom(n_pairs * 2, 1, 0.75),

cost_other_jpy = rgamma(n_pairs * 2, shape = 1.5, scale = 160) * rbinom(n_pairs * 2, 1, 0.6),

cost_antifungal_jpy = rgamma(n_pairs * 2, shape = 1, scale = 120) * rbinom(n_pairs * 2, 1, 0.3),

cost_antiviral_jpy = rgamma(n_pairs * 2, shape = 1, scale = 60) * rbinom(n_pairs * 2, 1, 0.25)

)

# --- 2.2: Calculate Average Daily Costs ---

total_cost_vars <- grep("^cost_", names(df), value = TRUE)

for (cost_var in total_cost_vars) {

avg_daily_cost_var <- gsub("cost_", "avg_daily_cost_", cost_var)

df[[avg_daily_cost_var]] <- ifelse(

is.na(df$days_to_discharge) | df$days_to_discharge == 0, 0,

df[[cost_var]] / df$days_to_discharge

)

}

# ==========================================================================

# SECTION 3: ANALYSIS OF CLINICAL OUTCOMES (Table 2 & Figure 2)

# ==========================================================================

cat("\n\n#################################################################")

cat("\n### SECTION 3: ANALYSIS OF CLINICAL OUTCOMES (Table 2 & Fig 2) ###")

cat("\n#################################################################")

# --- 3.1: Baseline Analysis (Table 2) ---

# Compares outcomes between Non-large (0) and Large (1) hospitals

cat("\n\n========== 3.1: Baseline Analysis (for Table 2) ==========\n")

# --- 3.1.1: Time to Discharge Alive (Table 2) ---

cat("\n--- Analyzing: Time to Discharge Alive (Baseline) ---\n")

baseline_cox <- coxph(

Surv(days_to_discharge, discharged_alive_indicator) ~ hospital_size_large + strata(matched_pair_id),

data = df

)

print(summary(baseline_cox))

# --- 3.1.2: 30-Day Mortality (Table 2) ---

cat("\n--- Analyzing: 30-Day All-Cause Mortality (Baseline) ---\n")

tryCatch({

baseline_mortality_clogit <- clogit(

mortality_30d ~ hospital_size_large + strata(matched_pair_id),

data = df

)

print(summary(baseline_mortality_clogit))

}, error = function(e) {

cat("ERROR in baseline mortality model:", e$message, "\n")

})

# --- 3.1.3: 30-Day Readmission (Table 2) ---

cat("\n--- Analyzing: 30-Day Unplanned Readmission (Baseline) ---\n")

tryCatch({

baseline_readmission_clogit <- clogit(

readmission_30d ~ hospital_size_large + strata(matched_pair_id),

data = subset(df, discharged_alive_indicator == 1) # Per protocol

)

print(summary(baseline_readmission_clogit))

}, error = function(e) {

cat("ERROR in baseline readmission model:", e$message, "\n")

})

# --- 3.2: Effect Modification Analysis (Figure 2) ---

cat("\n\n========== 3.2: Effect Modification Analysis (for Figure 2) ==========\n")

# --- 3.2.1: Helper function to get stratum-specific HR/OR ---

get_stratum_specific_cox_or_clogit <- function(model, effect_type = "OR") {

# (Function code remains the same as previous version)

coefs <- coef(model)

vcov_matrix <- vcov(model)

interaction_term_name <- "hospital_size_large:asp_fee_status"

b_non_large <- coefs["asp_fee_status"]

se_non_large <- sqrt(vcov_matrix["asp_fee_status", "asp_fee_status"])

b_large <- coefs["asp_fee_status"] + coefs[interaction_term_name]

se_large <- sqrt(

vcov_matrix["asp_fee_status", "asp_fee_status"] +

vcov_matrix[interaction_term_name, interaction_term_name] +

2 * vcov_matrix["asp_fee_status", interaction_term_name]

)

non_large_effect <- exp(c(b_non_large, b_non_large - 1.96 * se_non_large, b_non_large + 1.96 * se_non_large))

large_effect <- exp(c(b_large, b_large - 1.96 * se_large, b_large + 1.96 * se_large))

results <- rbind(`Non-large Hospitals` = non_large_effect, `Large Hospitals` = large_effect)

colnames(results) <- c(effect_type, "Lower 95% CI", "Upper 95% CI")

cat(paste("\n--- Stratum-Specific Effects of ASP Status ---\n"))

print(round(results, 3))

}

# --- 3.2.2: Time to Discharge Alive (Interaction) ---

cat("\n\n========== Analyzing: Time to Discharge Alive (Interaction) ==========\n")

interaction_cox <- coxph(

Surv(days_to_discharge, discharged_alive_indicator) ~ hospital_size_large * asp_fee_status + strata(matched_pair_id),

data = df

)

print(summary(interaction_cox))

get_stratum_specific_cox_or_clogit(interaction_cox, effect_type = "HR")

# --- 3.2.3 & 3.2.4: Mortality and Readmission (Interaction) ---

clinical_outcomes <- list("30-Day All-Cause Mortality" = "mortality_30d",

"30-Day Unplanned Readmission" = "readmission_30d")

for (outcome_name in names(clinical_outcomes)) {

cat(paste("\n\n========== Analyzing:", outcome_name, "(Interaction) ==========\n"))

outcome_var <- clinical_outcomes[[outcome_name]]

# Use subset for readmission analysis

analysis_data <- if (outcome_var == "readmission_30d") subset(df, discharged_alive_indicator == 1) else df

formula <- as.formula(paste(outcome_var, "~ hospital_size_large * asp_fee_status + strata(matched_pair_id)"))

tryCatch({

model <- clogit(formula, data = analysis_data)

# Check for infinite coefficients, a sign of complete separation

if (any(is.infinite(coef(model))) || any(is.na(coef(model)))) {

stop("Complete separation detected.")

}

print(summary(model))

get_stratum_specific_cox_or_clogit(model, effect_type = "OR")

}, warning = function(w){

cat("WARNING in model:", w$message, "\nThis may indicate model instability.\n")

}, error = function(e) {

cat("ERROR:", e$message, "\n")

cat("This is likely due to 'complete separation' (zero events in a subgroup).\n")

cat("Cross-tabulation of events:\n")

print(xtabs(as.formula(paste("~", outcome_var, "+ hospital_size_large + asp_fee_status")), data = analysis_data))

})

}

# =================================================================================

# SECTION 4: ANALYSIS OF ECONOMIC & PROCESS OUTCOMES (Table 3 & Figures 3, 4)

# =================================================================================

cat("\n\n##############################################################################")

cat("\n### SECTION 4: ANALYSIS OF ECONOMIC & PROCESS OUTCOMES (Table 3 & Figs 3, 4) ###")

cat("\n##############################################################################")

# --- 4.1: Baseline Analysis (Table 3) ---

# Compares outcomes between Non-large (0) and Large (1) hospitals

cat("\n\n========== 4.1: Baseline Analysis (for Table 3) ==========\n")

# --- 4.1.1: Analyze Duration of Antimicrobial Therapy (Baseline) ---

cat("\n\n--- Analysis: Antimicrobial Duration (Baseline) ---\n")

duration_outcomes <- c("duration_total", "duration_anti_mrsa", "duration_injectable", "duration_oral")

for (outcome in duration_outcomes) {

cat(paste("\n--- Outcome:", outcome, "---\n"))

formula <- as.formula(paste(outcome, "~ hospital_size_large + (1|matched_pair_id)"))

model <- glmmTMB(formula, data = df, family = nbinom2(link = "log"))

print(summary(model))

}

# --- 4.1.2: Analyze Average Daily Antimicrobial Costs (Baseline) ---

cat("\n\n--- Analysis: Average Daily Antimicrobial Costs (Baseline) ---\n")

cost_outcomes <- c("avg_daily_cost_total_jpy", "avg_daily_cost_anti_mrsa_jpy", "avg_daily_cost_antipseudomonal_jpy",

"avg_daily_cost_other_jpy", "avg_daily_cost_antifungal_jpy", "avg_daily_cost_antiviral_jpy")

for (outcome in cost_outcomes) {

cat(paste("\n--- Outcome:", outcome, "---\n"))

formula <- as.formula(paste(outcome, "~ hospital_size_large + (1|matched_pair_id)"))

tryCatch({

model <- glmmTMB(formula, data = df, family = tweedie(link = "log"), ziformula = ~0)

print(summary(model))

}, error = function(e) {

cat("ERROR in Tweedie model for", outcome, ":", e$message, "\n")

})

}

# --- 4.2: Effect Modification Analysis (Figures 3 & 4) ---

cat("\n\n========== 4.2: Effect Modification Analysis (for Figures 3 & 4) ==========\n")

# --- 4.2.1: Helper function to get stratum-specific Rate Ratios ---

get_stratum_specific_rrs <- function(model) {

# (Function code remains the same as previous version)

coefs <- fixef(model)$cond

vcov_matrix <- vcov(model)$cond

interaction_term_name <- "hospital_size_large:asp_fee_status"

b_non_large <- coefs["asp_fee_status"]

se_non_large <- sqrt(vcov_matrix["asp_fee_status", "asp_fee_status"])

b_large <- coefs["asp_fee_status"] + coefs[interaction_term_name]

se_large <- sqrt(

vcov_matrix["asp_fee_status", "asp_fee_status"] +

vcov_matrix[interaction_term_name, interaction_term_name] +

2 * vcov_matrix["asp_fee_status", interaction_term_name]

)

non_large_effect <- exp(c(b_non_large, b_non_large - 1.96 * se_non_large, b_non_large + 1.96 * se_non_large))

large_effect <- exp(c(b_large, b_large - 1.96 * se_large, b_large + 1.96 * se_large))

results <- rbind(`Non-large Hospitals` = non_large_effect, `Large Hospitals` = large_effect)

colnames(results) <- c("Rate Ratio", "Lower 95% CI", "Upper 95% CI")

cat(paste("\n--- Stratum-Specific Effects of ASP Status ---\n"))

print(round(results, 3))

}

# --- 4.2.2: Analyze Duration of Antimicrobial Therapy (Interaction) ---

cat("\n\n--- Analysis: Antimicrobial Duration (Interaction) ---\n")

# duration_outcomes is already defined in 4.1.1

for (outcome in duration_outcomes) {

cat(paste("\n--- Outcome:", outcome, "---\n"))

formula <- as.formula(paste(outcome, "~ hospital_size_large * asp_fee_status + (1|matched_pair_id)"))

model <- glmmTMB(formula, data = df, family = nbinom2(link = "log"))

print(summary(model))

get_stratum_specific_rrs(model)

}

# --- 4.2.3: Analyze Average Daily Antimicrobial Costs (Interaction) ---

cat("\n\n--- Analysis: Average Daily Antimicrobial Costs (Interaction) ---\n")

# cost_outcomes is already defined in 4.1.2

for (outcome in cost_outcomes) {

cat(paste("\n--- Outcome:", outcome, "---\n"))

formula <- as.formula(paste(outcome, "~ hospital_size_large * asp_fee_status + (1|matched_pair_id)"))

tryCatch({

model <- glmmTMB(formula, data = df, family = tweedie(link = "log"), ziformula = ~0)

print(summary(model))

get_stratum_specific_rrs(model)

}, error = function(e) {

cat("ERROR in Tweedie model for", outcome, ":", e$message, "\n")

})

}

# ==========================================================================

# SECTION 5: COMPUTATIONAL ENVIRONMENT

# ==========================================================================

cat("\n\n##############################################")

cat("\n### SECTION 5: COMPUTATIONAL ENVIRONMENT ###")

cat("\n##############################################\n")

print(sessionInfo())

# --- END OF SCRIPT ---
